# Supplementary material for: Integrative analysis of drug-gene signatures in human pluripotent stem cells reveals prazosin as a novel SQSTM1 regulator for ALS therapeutics
Source: Stem Cell Reports. 2026 Jun 25;21(7):102977. doi: 10.1016/j.stemcr.2026.102977 (PMC13385447; doi:10.1016/j.stemcr.2026.102977)
Supplement: Document S1. Figures S1–S7 and Tables S1–S3 [file mmc1.pdf]

Stem Cell Reports, Volume *21*

Supplemental Information

Integrative analysis of drug-gene signatures in human pluripotent stem cells reveals prazosin as a novel SQSTM1 regulator for ALS therapeutics

Florine Roussange, Jacqueline Gide, Johana Tournois, Michel Cailleret, Anne Boland, Christophe Battail, Jean-François Deleuze, Hélène Polvèche, Didier Auboeuf, Knut Brockmann, Edor Kabashi, Anca Marian, Lina El Kassar, Sophie Blondel, François Salachas, Gaëlle Bruneteau, Marc Peschanski, Cécile Martinat, and Sandrine Baghdoyan

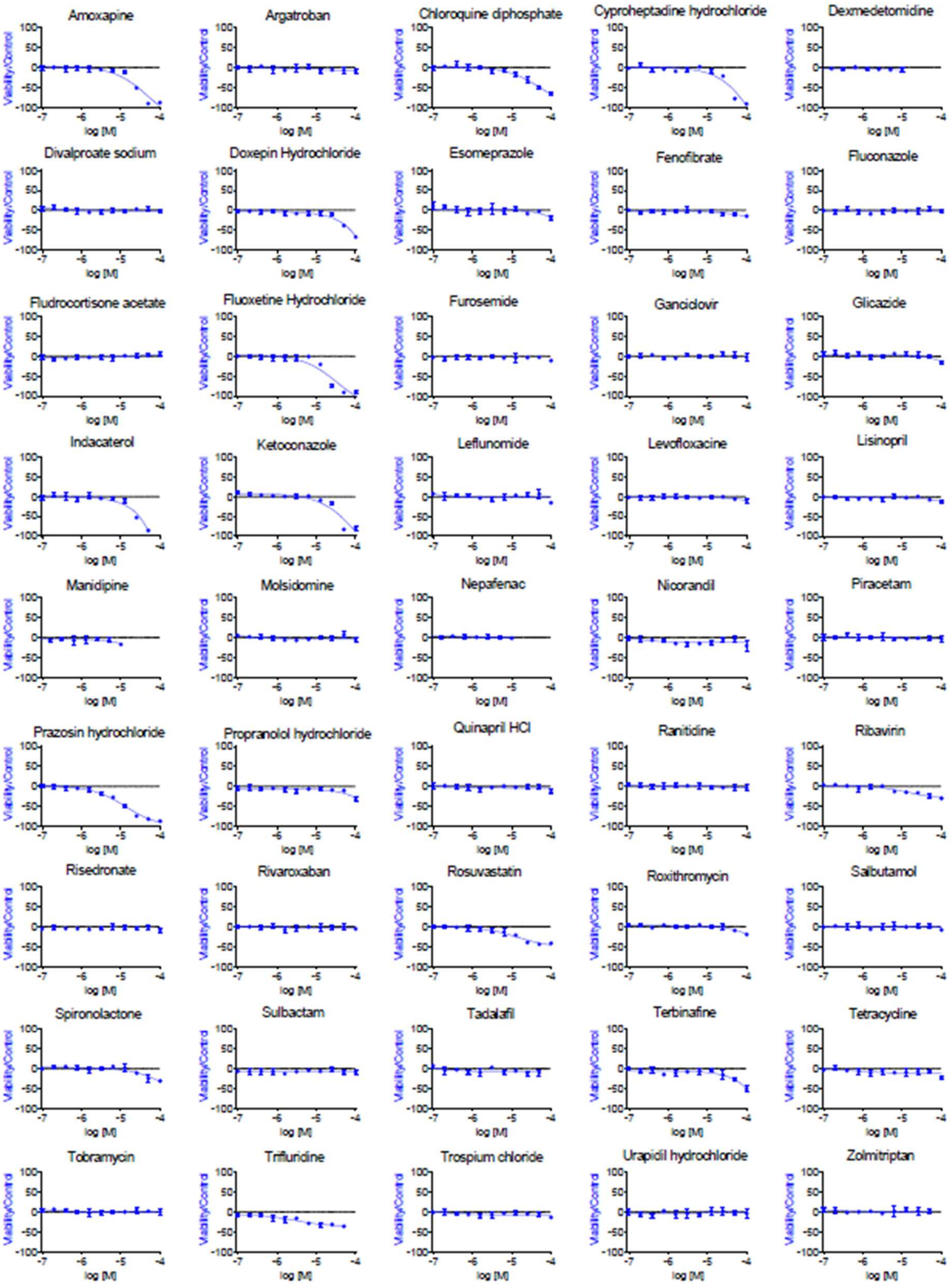

Amoxapine
Argatroban
Chloroquine diphosphate
Cyproheptadine hydrochloride
Dexmedetomidine
Divalproate sodium
Doxepin Hydrochloride
Esomeprazole
Fenofibrate
Fluconazole
Fludrocortisone acetate
Fluoxetine Hydrochloride
Furosemide
Ganciclovir
Glicazide
Indacaterol
Ketoconazole
Leflunomide
Levofloxacine
Lisinopril
Manidipine
Molsidomine
Nepafenac
Nicorandil
Piracetam
Prazosin hydrochloride
Propranolol hydrochloride
Quinapril HCl
Ranitidine
Ribavirin
Risedronate
Rivaroxaban
Rosuvastatin
Roxithromycin
Salbutamol
Spironolactone
Sulbactam
Tadalafil
Terbinafine
Tetracycline
Tobramycin
Trifluridine
Trospium chloride
Urapidil hydrochloride
Zolmitriptan
Viability/Control
100
50
0
-50
-100
-7
-6
-5
-4
log [M]

Figure S1. **Viability of hES-derived MPCs treated with the compounds of the smart repositioning library, related to Figure 1.** Cell viability of hES-derived MPCs was assessed using the Cell Titer-Glo assay following 24-hour treatment with various doses of drugs from the repositioning library.

A

		Nb of cell line with 2 < DE Z score > 2 / nb total of cell lines
Manumycin A	*LMNB1*	6/47
	SOD1	2/47
Rosuvastatin	*DESMIN*	2/13
	LMNB1	1/13
	PLP1	0/13
Prazosin	*PMP22*	0/13
	SQSTM1	4/13
Pentamidine	*EGR2*	non analyzed
	GDAP1	non analyzed
	PMP22	non analyzed

B

Gene	Mutation	Disease (OMIM)	Reference	Drug	DE FC in hES derived MSCs
MMP1	AR	RDEB (226600)	Titeux et al., 2008	Fludrocortisone acetate	-0,94
ITGB4	AR	JEB5A (619816)	Inoue et al., 2000	Rosuvastatin	3,28
DES	AD	MFM1 (601419)	Munoz-Marmol et al., 1998	Rosuvastatin	1,91
SQSTM1	AD	FTDALS3 (616437)	Fecto et al., 2011	Prazosin	2.43

C

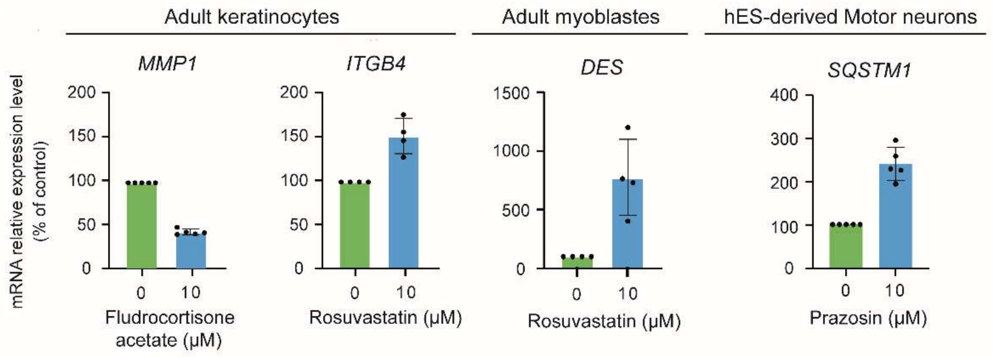

Figure S2. Analysis of gene expression regulation in various cellular contexts, related to Figure 1.

(A) Gene regulations validated by RT-qPCR in hES-derived MPCs were analyzed using the Broad Institute's Connectivity Map database.

(B) Identification of 4 genes - *MMP1* (matrix metallopeptidase 1), *ITGBA* (integrin subunit beta 4), *DES* (Desmin) and *SQSTM1* (Sequestosome 1) associated with a monogenic disease and responsive to treatment with drugs from the smart drug library.

(C) Plots of relative transcript expression for *MMP1, ITGB4, DES* and *SQSTM1* in human adult keratinocytes, human adult myoblasts and hES derived motor neurons (MNs) analyzed by RT-qPCR following 24-hour treatment with 10 µM of fludrocortisone acetate, rosuvastatin and prazosin. Data are presented as mean ± SD from three independent experiments.

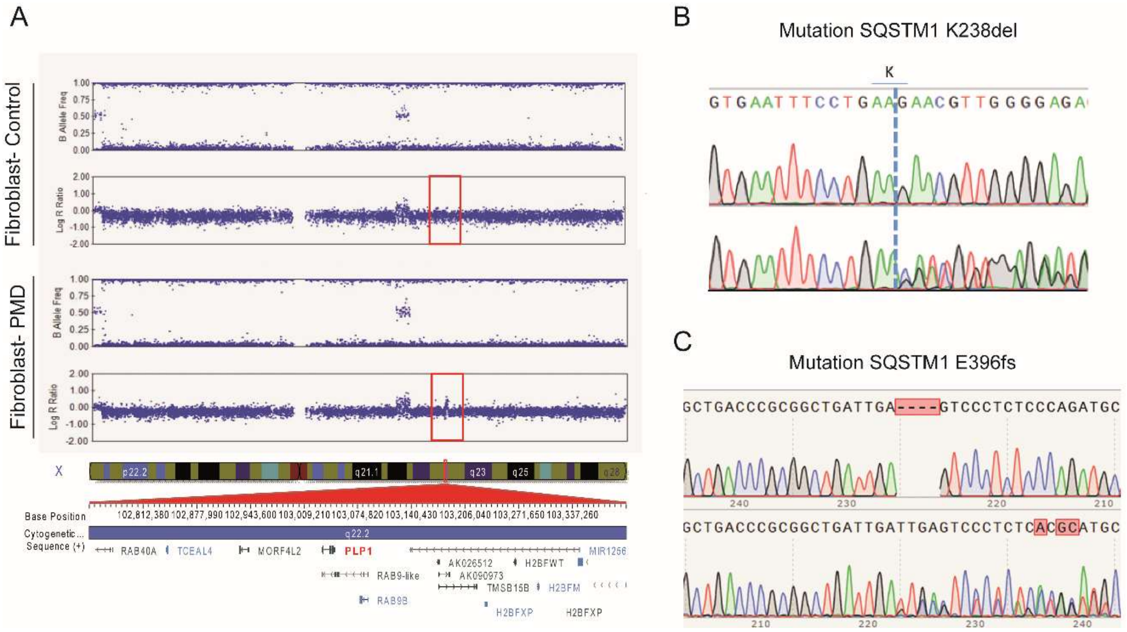

Figure S3. Identification of genetic alterations in pathological fibroblasts, related to Figure 2.

(A) SNP analysis of chromosome X in fibroblasts from non-affected or PMD affected patients.

(B and C) Sanger sequencing analysis of mutations in SQSTM1 K238del and SQSTM1 E396fs fibroblasts of FTD/ALS3 patients.

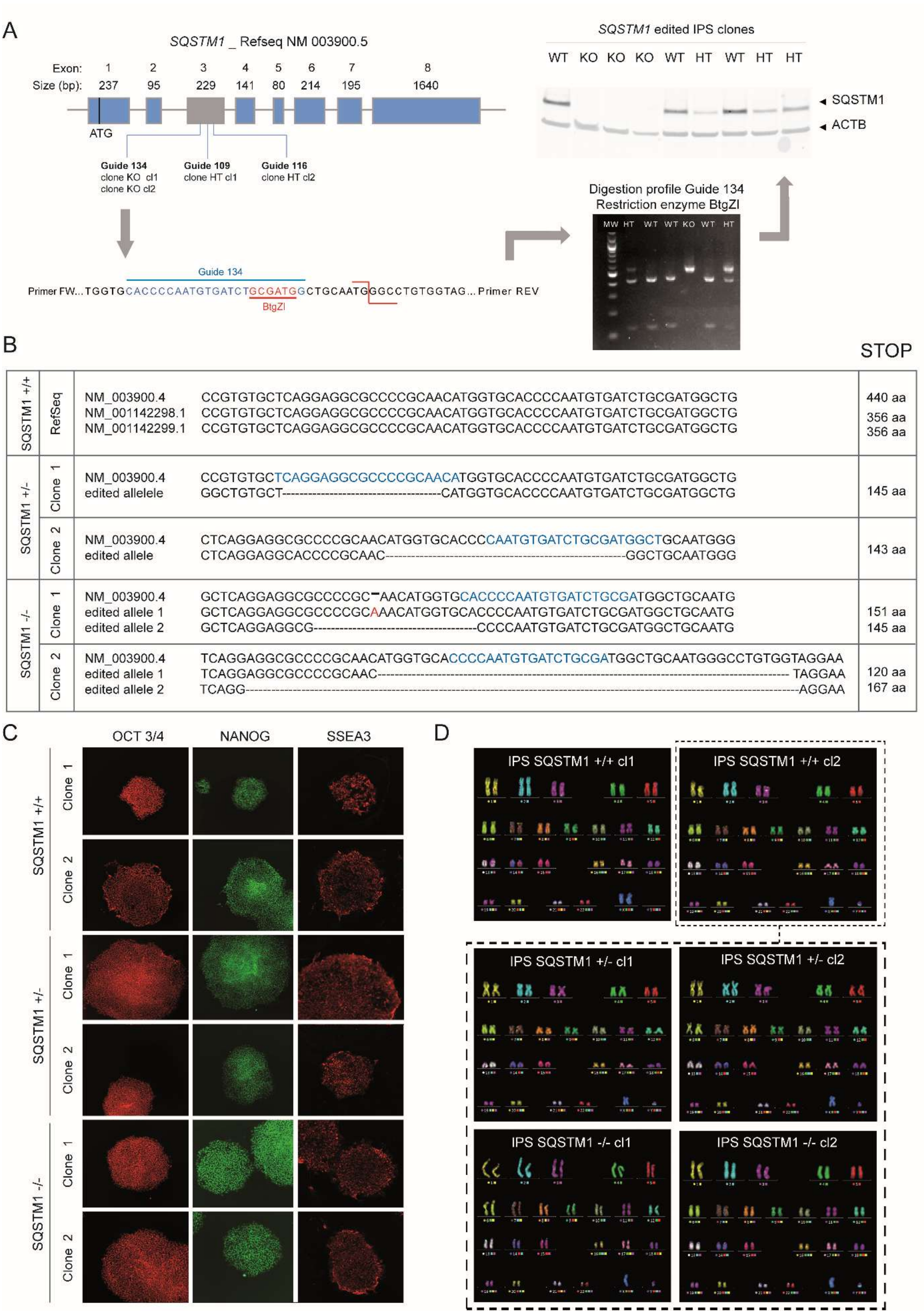

A
SQSTM1 _ Refseq NM 003900.5
Exon: 1 2 3 4 5 6 7 8
Size (bp): 237 95 229 141 80 214 195 1640
ATG
Guide 134
clone KO cl1
clone KO cl2
Guide 109
clone HT cl1
Guide 116
clone HT cl2
Guide 134
Primer FW...TGGTGCACCCCAATGTGATCTGCGATGGCTGCAATGGGCCTGTGGTAG...Primer REV
BtgZI
SQSTM1 edited IPS clones
WT KO KO KO WT HT WT HT HT
SQSTM1
ACTB
Digestion profile Guide 134
Restriction enzyme BtgZI
MW HT WT WT KO WT HT
B
STOP
SQSTM1 +/+
RefSeq
NM_003900.4 CCGTGTGCTCAGGAGGCGCCCCGCAACATGGTGCACCCCAATGTGATCTGCGATGGCTG 440 aa
NM_001142298.1 CCGTGTGCTCAGGAGGCGCCCCGCAACATGGTGCACCCCAATGTGATCTGCGATGGCTG 356 aa
NM_001142299.1 CCGTGTGCTCAGGAGGCGCCCCGCAACATGGTGCACCCCAATGTGATCTGCGATGGCTG 356 aa
SQSTM1 +/-
Clone 1
NM_003900.4 CCGTGTGCTCAGGAGGCGCCCCGCAACATGGTGCACCCCAATGTGATCTGCGATGGCTG
edited allelele GGCTGTGCT----------------------------------CATGGTGCACCCCAATGTGATCTGCGATGGCTG 145 aa
Clone 2
NM_003900.4 CTCAGGAGGCGCCCCGCAACATGGTGCACCCCAATGTGATCTGCGATGGCTGCAATGGG
edited allele CTCAGGAGGCACCCCGCAAC--GGCTGCAATGGG 143 aa
SQSTM1 -/-
Clone 1
NM_003900.4 GCTCAGGAGGCGCCCCGC-AACATGGTGCACCCCAATGTGATCTGCGATGGCTGCAATG
edited allele 1 GCTCAGGAGGCGCCCCGCAAACATGGTGCACCCCAATGTGATCTGCGATGGCTGCAATG 151 aa
edited allele 2 GCTCAGGAGGCG------------------------------------CCCCAATGTGATCTGCGATGGCTGCAATG 145 aa
Clone 2
NM_003900.4 TCAGGAGGCGCCCCGCAACATGGTGCACCCCAATGTGATCTGCGATGGCTGCAATGGGCCTGTGGTAGGAA
edited allele 1 TCAGGAGGCGCCCCGCAAC-- TAGGAA 120 aa
edited allele 2 TCAGG--AGGAA 167 aa
C
OCT 3/4
NANOG
SSEA3
SQSTM1 +/+
Clone 1
Clone 2
SQSTM1 +/-
Clone 1
Clone 2
SQSTM1 -/-
Clone 1
Clone 2
D
IPS SQSTM1 +/+ cl1
IPS SQSTM1 +/+ cl2
IPS SQSTM1 +/- cl1
IPS SQSTM1 +/- cl2
IPS SQSTM1 -/- cl1
IPS SQSTM1 -/- cl2

Figure S4. Generation of *SQSTM1* +/- and *SQSTM1* -/- hiPSC clones by the use of CRISPR/Cas9 technology, related to Figure 5.
(A) Schematic representation of the editing strategy targeting Exon 2 of the *SQSTM1* gene. Guide 134 was used to generate two different *SQSTM1* -/- clones from 14c5 hiPSCs, while guides 109 and 116 were employed to obtain *SQSTM1* +/- hiPSC clones 1 and 2, respectively. Edited clones were selected based on their restriction profiles, confirmed by SQSTM1 Western blot analysis.
(B) Sequences of *SQSTM1*-edited hiPSC clones show deletions or insertions on one or both alleles near the guide sequences (highlighted in blue). The position of the predicted premature STOP codon is indicated.
(C) Representative immunocytochemistry images showing pluripotency markers OCT3/4 (red), NANOG (green), and SSEA3 (red) staining in control and edited 1869 hiPSC colonies.
(D) Karyotype analysis of non-edited and edited *SQSTM1* hiPSC clones using mFish. *SQSTM1* +/- and *SQSTM1* -/- clones are derived from the hiPSC control clone 2 (cl2).

	Off-targets SQSTM1 -/- clones 1&2 sgRNA 134	CFD Score
1	intron_B4GALT7_chr5_177603242	0,45
2	intron_FAM184A_chr6_119129291	0,32
3	intergenic_LINC00459|LINC00448_chr13_62397889	0,31
4	intergenic_RP11-420N3.3/RP11-420N3.2|RP11-420N3.2/RP11-420N3.3_chr16_5491023	0,29
5	intron_TESC_chr12_117069990	0,24
6	intergenic_LINC00379|MIR17HG_chr13_91300197	0,24
7	intergenic_NPPA|NPPB_chr1_11854812	0,24
8	intergenic_RP11-403A3.3|LINC00396_chr13_110042906	0,23
9	intergenic_RP11-520A21.1|RP11-372H2.1_chr3_41033547	0,23
10	intron_DIAPH2_chrX_97220513	0,22

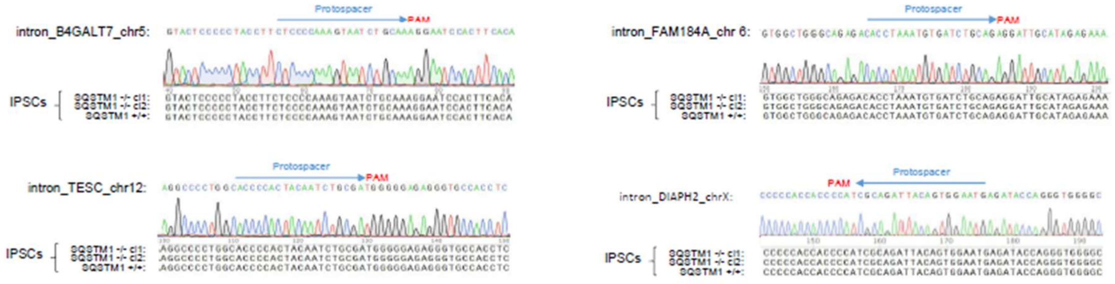

	Off-targets SQSTM1 +/- clone 1 sgRNA 109	CFD Score
1	intergenic_PRICKLE2|RP11-14D22.2/PRICKLE2_chr3_64350556	0.58
2	intron_DNAH17_chr17_78426708	0.25
3	intergenic_LINC00320|NCAM2_chr21_20905590	0.24
4	intergenic_LL09NC01-139C3.1|RP11-145E17.2_chr9_134240780	0.24
5	intergenic_LINC00961|RP11-327L3.3_chr9_35914472	0.20
6	intergenic_CCL1|AC011193.1_chr17_34424483	0.19
7	intergenic_RNA5SP167|RNU6-1230P_chr4_149916252	0.19
8	intergenic_C17orf74|TMEM102_chr17_7432704	0.17
9	intron_RP11-218F4.1_chr17_27963596	0.16
10	intergenic_CTD-2316B1.2|TMEM161B_chr5_88053056	0.13

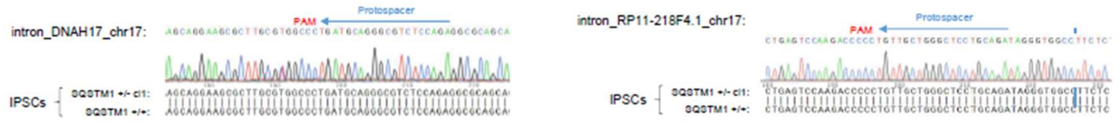

	Off-targets SQSTM1 +/- clone 2 sgRNA 116	CFD Score
1	intergenic_RP11-428L9.2|LINC00709_chr10_9174267	0.69
2	intergenic_GSK3B|AC092910.1_chr3_120041470	0.65
3	intron_CD109_chr6_73750302	0.43
4	intron_MYT1_chr20_64172097	0.42
5	intron_FAM114A1_chr4_38925695	0.38
6	intron_NABP1_chr2_191685192	0.35
7	intergenic_RP11-384F7.1|IGSF11_chr3_118619727	0.31
8	intergenic_RP11-100M12.2|AC104002.1_chr15_27429789	0.26
9	intergenic_RP11-420N3.3/RP11-420N3.2|RP11-420N3.2/RP11-420N3.3_chr16_5491025	0.22
10	intergenic_AL356154.1|NRG3_chr10_81149806	0.21

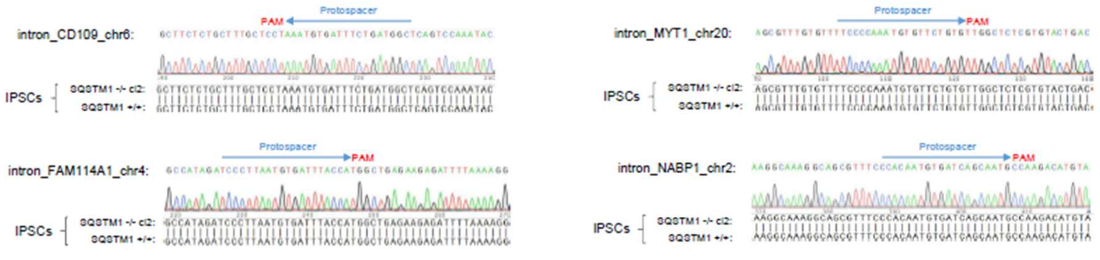

Figure S5. Off target analysis in edited *SQSTM1* +/- and *SQSTM1* -/- hiPSC lines, related to Figure 5. Potential off target edition sites in genomic DNA were identified with the CRISPOR (http://crispor.tefor.net/) software for the three guides used in the study. Region of interest located within genes were amplified by PCR and analyzed by Sanger sequencing. CFD means Cutting frequency determination indicated by the CRISPOR software.

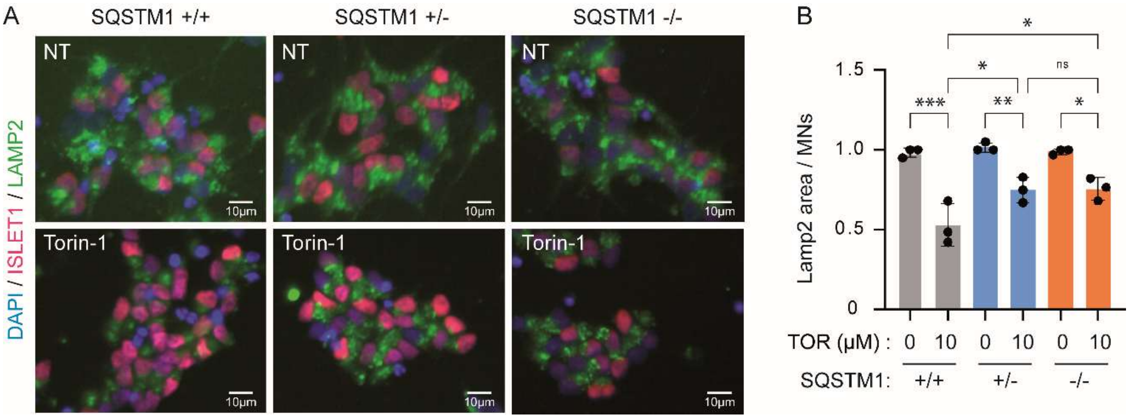

Figure S6. Impact of SQSTM1 loss of expression on LAMP2⁺ lysosomes after Torin-1 treatment in hiPSCs derived MNs, related to Figure 5.

(A and B) Representative immunocytochemistry images (A) and quantification (B) of DAPI (blue), ISLET1 (red), and LAMP2 (green) staining in hiPSC-derived MNs with *SQSTM1*+/+, *SQSTM1*+/-, and *SQSTM1*-/- genotypes treated with 10 µM Torin-1 for 24 hours. Scale bar: 10µm. Plots display the LAMP2 staining area normalized to ISLET1+ MNs, presented as mean ± SD from three independent differentiations from MNs progenitors. Statistical significance was determined using two-way ANOVA with Šídák's post hoc multiple comparisons test.

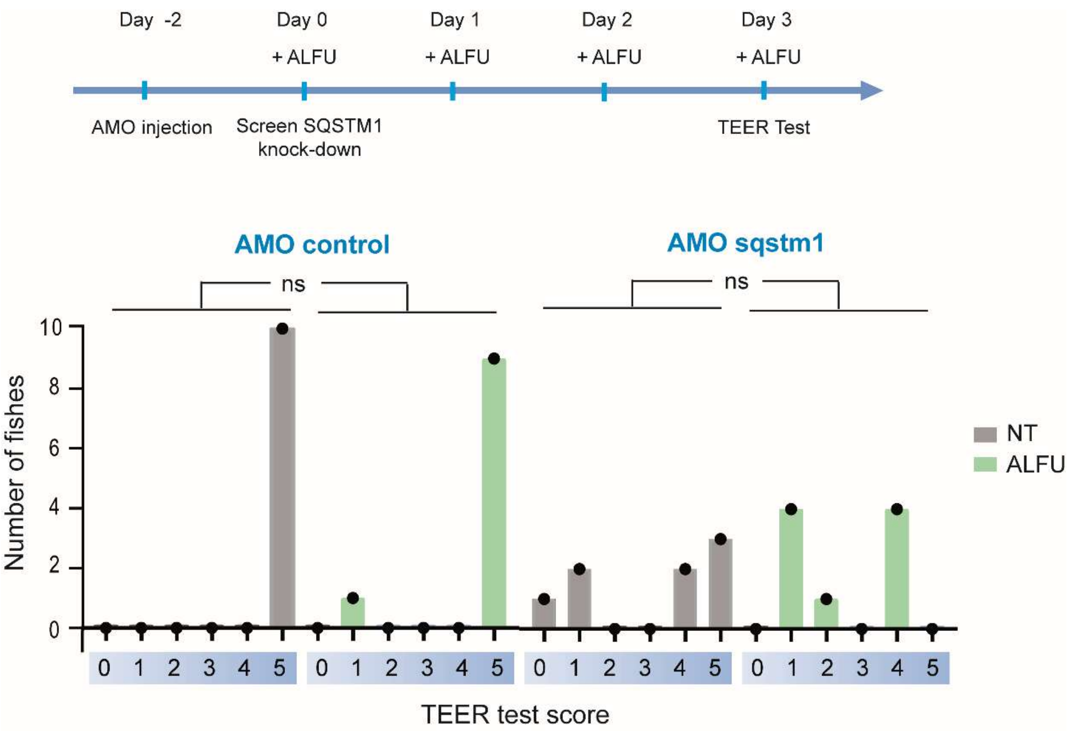

Figure S7. Analysis of alfuzosin treatment on zebrafish with sqstm1 knock-down, related to Figure 7. Quantification of swimming parameters in embryos injected with control or sqstm1-specific AMO, either untreated or treated with alfuzosin for 3 days, using a ViewPoint system. The qualitative scores ranged from 0-5. Data are presented from one experiment on 37 zebrafishes. Statistical significance was determined using a Chi-square test for contingency data. ns: not significative.

Table S1. List of the Drugs tested for their repositioning potential, related to Figure 1.

Drug	Synonyms	Activity	Family/Therapeutic target	Pubchem CID	FDA or EMA-approved	French AMM
Cyproheptadine	Periactine	Anti-Allergic	Serotonin receptor antagonist	2913	+	
Molsidomine	Corvasal	Anti-Anginal	Relaxation in the coronary blood vessels	5353788	-	+
Nicorandil	Ikorel	Anti-Anginal	Arterial and a venous dilator	47528	-	+
Indacaterol	Onbreezhaler	Anti-Asthmatic	Bronchodilator	6918554	+	
Salbutamol	Albuterol	Anti-Asthmatic	Bronchodilator	2083	+	
Levofloxacine	Tavanique	Anti-Bacterial	Fluoroquinolone antibiotic	149096	+	
Manumycin	Manumycin A	Anti-Bacterial	Fatty amide	6438330	-	-
Roxithromycin	Rulid	Anti-Bacterial	Macrolide antibiotic	6915744	-	+
Sulbactam	Unasyn	Anti-Bacterial	Beta-lactamase inhibitor	130313	+	
Tetracycline	Pylera	Anti-Bacterial	polyketide antibiotic	54675776	+	
Tobramycin	Tobradex	Anti-Bacterial	Aminoglycoside antibiotic	36294	+	
Argatroban	Arganova	Anti-Coagulant	Platelet Aggregation Inhibitors	92722	+	
Rivaroxaban	Xarelto	Anti-Coagulant	Inhibitor of the coagulation factor Xa	9875401	+	
Piracetam	Nootropyl	Anti-Convulsant	Neurotransmission promoter	4843	-	+
Amoxapine	Defanyl	Anti-Depressant	Norepinephrine and serotonin re-uptake inhibitor	2170	+	
Doxepin	Quitaxon	Anti-Depressant	Norepinephrine and serotonin-reuptake inhibitor	3158	+	
Fluoxetine	Prozac	Anti-Depressant	Selective serotonin-reuptake inhibitor	3386	+	
Gliclazide	Diamicron	Anti-Diabetic	Insulin secretagogue	3475	-	+
Glyburide	Daonil	Anti-Diabetic	Insulin secretagogue	3488	+	
Pioglitazone	Actos	Anti-Diabetic	Inducer of cellular responsiveness to insulin	4829	+	
Divalproate sodium	Depakote	Anti-Epileptic	Gamma-aminobutyric acid (GABA) transaminase inhibitor	18330669	+	
Fluconazole	Diflucan	Anti-Fungal	Triazole	3365	+	
Ketoconazole	Ketoderm	Anti-Fungal	Phenylpiperazine	456201	+	
Pentamidine	Pentacarinat	Anti-Fungal	Interfering with DNA replication	4735	+	
Terbinafine	Lamisil	Anti-Fungal	Equalene epoxidase inhibitor	1549008	+	
Furosemide	Lasilix	Anti-Hypertensive	Potent loop diuretic	3440	+	
Lisinopril	Zestril	Anti-Hypertensive	Angiotensin-converting enzyme (ACE) inhibitor	5362119	+	
Manidipine	Iperten	Anti-Hypertensive	calcium channel blocker	4008	-	+
Prazosin	Minipress	Anti-Hypertensive	Alpha-1 adrenergic receptor inhibitor	4893	+	
Propranolol hydrochloride	Avlocardyl	Anti-Hypertensive	Beta-adrenergic receptor blocker	62882	+	
Quinapril HCl	Accupril	Anti-Hypertensive	Angiotensin converting enzyme (ACE) inhibitor	54891	+	
Spironolactone	Aldactone	Anti-Hypertensive	Potassium-sparing diuretic	5833	+	
Tadalafil	Cialis	Anti-Hypertensive	Cyclic guanosine monophosphate (cGMP)-specific type 5 phosphodiesterase inhibitor	110635	+	
Urapidil	Eupressyl	Anti-Hypertensive	Adrenergic alpha-1 receptors inhibitor	5639	-	+
Risedronic sodium	Actonel	Anti-Hypocalcemic	Bisphosphonate	5245	+	
Fludrocortisone acetate	Flucortac	Anti-inflammatory	Glucocorticoid-receptor agonist	225609	+	
Indoprofen	Flosint	Anti-inflammatory	Prostaglandin-endoperoxide synthase inhibitor	3718	-	-
Leflunomide	Arava	Anti-inflammatory	Antirhumatismal	3899	+	
Nepafenac	Nevanac	Anti-inflammatory	Cyclooxygenase 1 and 2 inhibitor	151075	+	
Fenofibrate	Lipanthyl	Anti-Lipidemic	Peroxisome proliferator activated receptor alpha (PPARalpha) activator	3339	+	
Rosuvastatin	Crestor	Anti-Lipidemic	Hydroxymethyl-glutaryl coenzyme A (HMG-CoA) reductase inhibitor	446157	+	
Chloroquine	Plaquenil	Anti-Malarial	Inhibit the parasitic enzyme heme polymerase	2719	+	
Zolmitriptan	Zomig	Anti-Migraine	serotonin (5-HT) 1B receptors activator	60857	+	
Esomeprazole	Innexium	Anti-Peptic	Gastric proton pump inhibitor	9568614	+	
Ranitidine	Azantac	Anti-Peptic	Histamine H2-receptor antagonists	3001055	+	
Trospium chloride	Ceris	Anti-Spasmotic	Muscarinic receptors blockade	5284632	+	
Ganciclovir	Virgan	Anti-Viral	Analogue nucleoside	1,35E+08	+	
Ribavirin	Rebetol	Anti-Viral	Viral RNA synthesis inhibitor	37542	+	
Trifluridine	Viroptic	Anti-Viral	thymidylate synthase inhibitor	6256	+	
Dexmedetomidine	Dexdor	Sedative	Alpha-adrenergic agonist	5311068	+	

Table S2. Differentially expressed genes and splicing after drug treatment in MPCs, related to Figure 1.

Drug	DESeq log2FC ≥ 1,45			DESeq log2FC ≥ 0,4			Farline
	DESeq up	DESeq down	Total	DESeq up	DESeq down	Total	
**DMSO**	x	x	x	x	x	x	x
Amoxapine	19	0	19	279	144	423	54
Argatroban	0	0	0	0	1	1	44
Chloroquine	31	1	32	758	383	1141	17
Cyproheptadine	11	0	11	312	89	401	54
Dexmedetomidine	0	0	0	0	0	0	27
Divalproate sodium	0	0	0	0	7	7	73
Doxepin	4	0	4	60	1	61	0
Esomeprazole	0	0	0	0	0	0	30
Fenofibrate	0	0	0	0	0	0	34
Fluconazole	0	0	0	0	0	0	31
Fludrocortisone acetate	3	0	3	108	162	270	31
Fluoxetine	17	0	17	239	93	332	32
Furosemide	0	0	0	0	0	0	47
Ganciclovir	0	0	0	0	0	0	15
Gliclazide	0	0	0	0	0	0	46
Glyburide	0	0	0	0	9	9	53
Indacaterol	24	0	24	385	132	517	47
Indoprofen	0	0	0	0	7	7	29
Ketoconazole	11	0	11	143	21	164	27
Leflunomide	0	0	0	25	38	63	21
Levofloxacine	0	0	0	0	0	0	23
Lisinopril	0	0	0	0	0	0	12
Manidipine	2	0	2	145	68	213	45
Manumycin	236	217	453	1508	1517	3025	125
Molsidomine	0	0	0	0	0	0	31
Nepafenac	0	0	0	0	3	3	36
Nicorandil	0	0	0	0	0	0	18
Pentlseth	124	128	252	1459	1446	2905	206
Pioglitazone	0	0	0	0	0	0	21
Piracetam	0	0	0	0	0	0	22
Prazosin	138	15	153	1418	1209	2627	70
Propranolol hydrochloride	0	0	0	0	0	0	27
Quinapril HCl	0	0	0	0	0	0	20
Ranitidine	0	0	0	0	0	0	10
Ribavirin	0	0	0	24	38	62	75
Risedronic acid	0	0	0	0	0	0	25
Rivaroxaban	0	0	0	7	11	18	48
Rosuvastatin	182	306	488	1724	1947	3671	140
Roxithromycin	0	0	0	17	3	20	17
Salbutamol	0	0	0	6	1	7	27
Spironolactone	0	0	0	46	41	87	51
Sulbactam	0	0	0	0	0	0	0
Tadalafil	0	0	0	1	0	1	44
Terbinafine	0	0	0	0	0	0	16
Tetracycline	0	0	0	0	0	0	47
Tobramycin	0	0	0	0	0	0	21
Trifluridine	3	0	3	348	59	407	57
Trospium chloride	0	0	0	0	0	0	34
Urapidil	0	0	0	0	0	0	49
Zolmitriptan	0	0	0	0	8	8	42

Table S3. List of the primers and guides used in the study, related to Figure 1, Figure 2, Supplemental Figure 2, Figure 3, Figure 5 and Figure 7.

**Primers for Gene expression analysis**		
Gene	Primer Forward	Primer Reverse
PMP22	GCTCCTCCTGTTGCTGAGTA	ATCGACAGGATCATGGTGGC
EGR2	ACCGCCTCCTCCTCCTTATT	GGGTAGGCCAGAGAGGAAGA
DES	ATTGGAGGACCGATTTGCC	TCACCGTCTTCTTGGTATGGA
LMNB1	AGCTGCAAATCTGATGGCCT	GAGGAACCCCTTCCGAACAG
SOD1	TTGGGCAAAGGTGGAAATGAA	CACCACAAGCCAAACGACTT
GDAP1	GACTCCATGATCCCGGCTTA	ACCTGATCCAAGACTTTCTCCA
SQSTM1	AGGACAAATTGCGCCCATTT	TCTCTTTCAGGGACAGGCTG
PLP1	TGAAGCCCTAACTCAGCCAA	AGGGCCATCTCAGGTTACAC
18S	GAGGATGAGGTGGAACGTGT	TCTTCAGTCGCTCCAGGTCT
HSPB8	AAATGTTAGAGGGTGCGGG	TGGCCTAACACAACCCAAGCA
HSPA1A	AGCTGGAGCAGGTGTGTAAC	CAGCAATCTTGGAAAGGCCC
HSPA1B	AGC TGG AGC AGG TGT GTA AC	GAG TCC CAA CAG TCC ACC TC
HSPA5	GAACGTCTGATTGGCGATGC	ACCACCTTGAACGGCAAGAA
HSPH1	AGCAGAAATGAAGTTAGTGTCCA	TGTTTTCTTCTGGGTGGCAAG
OPTN	GACCAGCAGGCTTACCTTGT	GGGGCAGGAATGAATCGGAA
UBC	AACAACAACTGCGACACCCAAA	GGAACAGGCGAGGAAAAGTA
MAP1LC3B	CCGCACCTTCGAACAAAGAG	TTGAGCTGTAAGCGCCTTCT
ITGB4	AATGCAGCCGGTCTGACTC	GTCCTGGACCTACCTCGGG
MMP1	CTGCTGGCAAAACATCCCTT	CGCTTTGATGGCATTGAGGA

**Primers for Offtarget analysis in SQSTM1 edited iPSC clones**				
SQSTM1 edited clone	Guide Sequences	off target	Forward Primer	Reverse Primer
SQSTM1 -/- clones 1&2 sgRNA 134	TCAGGAGGCGCCCCGCAACA TGG	intron_B4GALT7_chr5_177603242	TCGCTGACTCTTAAGATTGTGA	CGCTCCTCTGGAAGTTCCTG
		intron_FAM184A_chr6_119129291	ACCTATAACCTGGAAGCCCC	CCCACCACATCTGCTTCATG
		intron_TESC_chr12_117069990	TGGCACATCTTGGAAGCTG	AAGCCTGTGGTTCTGAATGA
		intron_DIAPH2_chrX_97220513	CTGTTCCACCATCTGCTCCC	ATTTTACCACGGCCCCTCTG
SQSTM1 +/- clones 1 sgRNA 109	AGCCATCGCAGATCACATTG GGG	intron_DNAH17_chr17_78426708	AGAGAGCCGTGGACAGATCT	CTCAAGGAGCTGAACCTGGG
		intron_RP11-218F4.1_chr17_279635	AAGTGGGGCCTGCATAAGTC	TGCAGGAAAGATGGGCTGAG
SQSTM1 +/- clones 2 sgRNA 116	CACCCCAATGTGATCTGCGA TGG	intron_CD109_chr6_73750302	AGCTCAGTTTGGGTGAGTGG	ATATTGCTGCTGCCTCCACC
		intron_MYT1_chr20_64172097	CTCTTCCTGGAGAGCCCTCT	AGAAGGGAGAGAGCTCGAGG
		intron_FAM114A1_chr4_38925695	AAATCCAGGAGCAATCCAGC	CTGCAACAAACGTGAAAGTGT
		intron_NABP1_chr2_191685192	TGACTCGAAGGCTACATGTCT	CCTTGATTCAGGGCCAGTGT
