# Supplementary material for: Integrative analysis of drug-gene signatures in human pluripotent stem cells reveals prazosin as a novel SQSTM1 regulator for ALS therapeutics
Source: Stem Cell Reports. 2026 Jun 25;21(7):102977. doi: 10.1016/j.stemcr.2026.102977 (PMC13385447; doi:10.1016/j.stemcr.2026.102977)
Supplement: Document S2. Article plus supplemental information [file mmc2.pdf]

STEM CELL REPORTS
Article
OPEN ACCESS

Integrative analysis of drug-gene signatures in human pluripotent stem cells reveals prazosin as a novel SQSTM1 regulator for ALS therapeutics

Florine Roussange,[1] Jacqueline Gide,[2,11] Johana Tournois,[3] Michel Cailleret,[1] Anne Boland,[4] Christophe Battail,[4,5] Jean-François Deleuze,[4] Hélène Polvèche,[3] Didier Auboeuf,[6] Knut Brockmann,[7] Edor Kabashi,[8] Anca Marian,[8] Lina El Kassar,[3] Sophie Blondel,[2] François Salachas,[9] Gaëlle Bruneteau,[9,10] Marc Peschanski,[2] Cécile Martinat,[1,2,12,*] and Sandrine Baghdoyan[1]
[1]Université Paris Saclay, Université d'Evry, Inserm, IStem, UMR861, 91100 Corbeil-Essonnes, France
[2]IStem, CECS, 91100 Corbeil-Essonnes, France
[3]IStem, CECS, the research and innovation team, 91100 Corbeil-Essonnes, France
[4]Université Paris-Saclay, CEA, Centre National de Recherche en Génomique Humaine (CNRGH), 91057 Evry, France
[5]Université Grenoble Alpes, Inserm, CEA, UA13, BGE, 38000 Grenoble, France
[6]Ecole Normale Supérieure de Lyon, Inserm, U1293, CNRS, UMR 5239, Université Claude Bernard Lyon 1, Laboratory of Biology and Modelling of the Cell, 46 allée d'Italie 69364 Lyon, France
[7]Department of Pediatrics and Adolescent Medicine, University Medical Center, Göttingen, Germany
[8]Laboratory of Translational Research for Neurological Disorders, Imagine Institute, Université de Paris, INSERM, UMR 1163, 75015 Paris, France
[9]Sorbonne Université, Institut du Cerveau - Paris Brain Institute - ICM, APHP, Inserm, CNRS, Département de Neurologie, Centre SLA de Paris, Hôpital Pitié-Salpêtrière, 75013 Paris, France
[10]Alliance on Clinical Trials for ALS-MND (ACT4ALS-MND), Neuroscience Clinical Investigation Center, Paris Brain Institute, 75013 Paris, France
[11]Deceased
[12]Lead contact
*Correspondence: cmartinat@istem.fr
https://doi.org/10.1016/j.stemcr.2026.102977

SUMMARY

The classical paradigm of drug screening often faces significant limitations due to the challenges associated with identifying molecular or cellular read-outs that are relevant to specific genetic diseases. To remedy this, an alternative approach of reverse phenotypic mapping was tested: Compounds were evaluated for their effects on gene expression and alternative splicing in a healthy cell model, and the resulting data were matched to molecular signatures of diseases. A subset of 50 drugs was tested on mesenchymal stem cells derived from a human pluripotent stem cell line. Over half of the compounds altered gene expression, many affecting pathways linked to monogenic diseases. One hit, increased SQSTM1 expression induced by prazosin, was further validated in FTD/ALS type 3 models caused by SQSTM1 haploinsufficiency, including patient-derived fibroblasts, SQSTM1-depleted hiPSC-derived motor neurons, and a zebrafish model. Extending this paradigm could involve testing diverse cell types and larger drug libraries.

INTRODUCTION

Drug discovery has long been the cornerstone of medical progress, traditionally focused on testing a wide range of pharmacological compounds against a predefined target, or "readout," linked to a specific disease. While this paradigm has achieved notable successes, it faces significant limitations, mainly due to its single-target approach to addressing a specific pathology (Sertkaya et al., 2024; Vincent et al., 2022). As a result, the scope of discovery is largely confined to the interaction between a drug and a target, often under highly controlled conditions, which limits the consideration of the wider biological context. Indeed, the diversity in classical drug screening depends largely on the pharmacological compounds tested and their application parameters (e.g., dosage, timing). This fails to fully exploit the rich biological variability within cellular systems and molecular mechanisms, critical factors especially for diseases driven by complex changes in gene expression and regulatory networks. Beyond these issues, classical drug screening faces difficulties in effectively exploiting the wealth of data provided by modern genomic and transcriptomic databases. Advances in high-throughput sequencing technologies and computational resources have generated vast datasets cataloging gene expression profiles and splicing variations in many diseases (Cummings et al., 2017; R. Wang et al., 2023). These data provide valuable information that could significantly improve drug discovery if properly integrated into screening strategies. Initiatives such as the Broad Institute's Connectivity Map have begun to generate comprehensive resources for analyzing gene expression changes in various tumor cell types in response to specific compounds (Lamb et al., 2006; Subramanian et al., 2017). This widely used database has been instrumental in identifying potential agents that target key cancer-related pathways (Dong et al., 2021; Fortney et al., 2015; Gonzalez-Fierro and Duenas-Gonzalez, 2021; Zhao et al., 2023). Such an approach offers significant potential for addressing rare genetic diseases, where a single gene mutation or a well-defined pathway is often at the root of the condition. By focusing on the cell types most pertinent to these specific pathologies, it becomes possible to target more relevant therapeutic mechanisms. The effectiveness of this

© 2026 The Authors.
Published by Elsevier Inc. on behalf of International Society for Stem Cell Research.
This is an open access article under the CC BY license (http://creativecommons.org/licenses/by/4.0/).

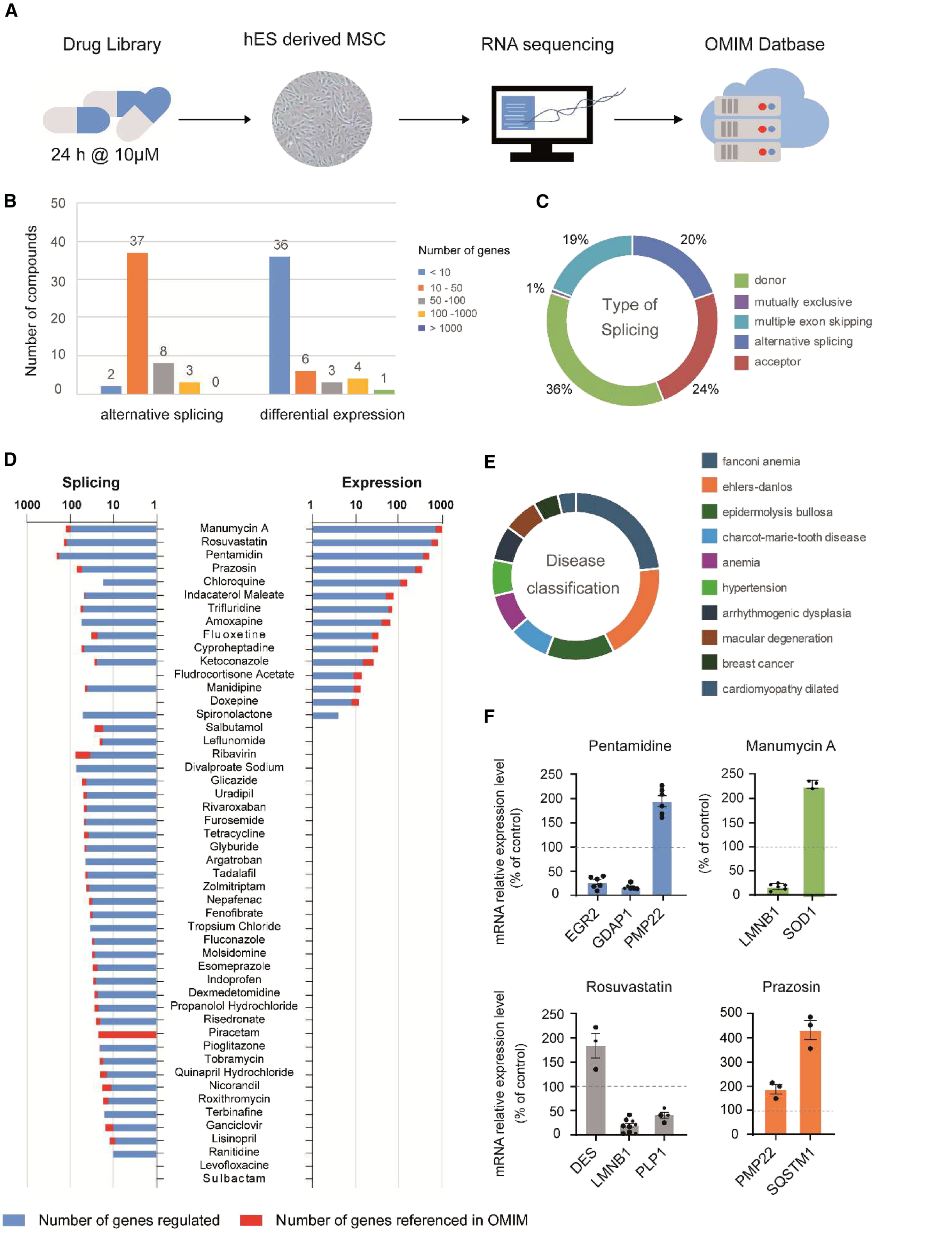

(legend on next page)

strategy can be further amplified when combined with repositionable drugs—compounds that have already been approved by regulatory agencies for other diseases—speeding up the drug development process and increasing the likelihood of finding effective therapies (Roessler et al., 2021). However, to take full advantage of this potential, it is necessary to change the paradigm of drug discovery. Rather than focusing solely on a specific disease, an agnostic approach to drug discovery could be adopted, whereby compounds are tested first and then the observed changes in gene expression and splicing are linked to relevant diseases.

In this study, we explored such an alternative approach to the classical drug screening paradigm by evaluating the potential of integrating human pluripotent stem cell (hPSC) derivatives with deep RNA sequencing to map gene expression signatures induced by 50 compounds, most of which are drugs approved in the US, Europe, or Asia. More than half of the compounds influenced gene expression, and many affected pathways linked to monogenic diseases. To assess the predictive value of this approach, we focused on prazosin, an antihypertensive drug identified as a modulator of *SQSTM1* expression. *SQSTM1* encodes the autophagy receptor SQSTM1/p62, mutations in which are linked to rare forms of amyotrophic lateral sclerosis (Fecto et al., 2011; Renton et al., 2014). Using patient-derived fibroblasts, SQSTM1-depleted hiPSC-derived motor neurons (MNs), and an *in vivo* zebrafish model with *sqstm1* knockdown, we demonstrated that prazosin treatment can enhance autophagy-related processes and improve MN phenotypes, correlating with improved motor behavior *in vivo*.

RESULTS

Gene expression signature induced by 50 biologically active small molecules

As a proof of concept, we selected a panel of 50 small molecules, including 41 drugs approved in the US and Europe, based on (1) high prescription frequency, (2) established safety profiles, and (3) chemical and biological diversity (Table S1). Drug effects were evaluated in wild-type mesodermal progenitor cells (MPCs) derived from human embryonic stem cells, chosen for their ease of production, cost-effectiveness, and demonstrated suitability for drug screening applications (Maury et al., 2019) (Figure 1A). Each compound was tested at a single concentration of 10 μM for 24 h, a dose determined not to induce more than 50% cytotoxicity (Figure S1). Transcriptomic responses were assessed using next-generation sequencing. This experimental condition, which was also used in the Broad Institute's Connectivity Map study, will enable a comparison to be made between our dataset and those obtained from other cell types of the study. The potential of the 50 compounds was evaluated by analyzing their capacity to influence both transcript expression and alternative splicing. The percentage of alternative spliced values was estimated by systematic comparison of a mapping-first approach (FARLINE) (Benoit-Pilven et al., 2018) using a threshold of at least 10% change of the mean value. Analysis of ΔPSI (percent spliced In) values revealed that the majority of compounds (74%) triggered fewer than 50 splicing events (Figure 1B; Table S2), suggesting that most drugs induce relatively modest alterations in splicing patterns. These changes affected a wide range of RNA splicing types, with the notable exception of mutually exclusive exon events, which accounted for only 1% of cases (Figure 1C). Three compounds, manumycin A, pentamidine and rosuvastatin, emerged as having notable regulatory effects, each affecting more than 100 splicing events. Two of these drugs, namely manumycin A and pentamidine, had previously been identified as alternative splicing regulators with therapeutic potential for treating spliceopathies (Oana et al., 2013; Parkesh et al., 2012). Although 88% of the compounds inducing splicing alterations were found to target genes associated with genetic diseases (Table S2), no therapeutic effect could be directly linked to exon skipping events involving clinically relevant SNP variants. Differentially expressed genes (DEGs) were identified using a threshold of false discovery rate (FDR) < 0.05 and

Figure 1. Molecular screening of repositioning drugs by RNA-seq identifies new therapeutic candidates for rare diseases

(A) Schematics illustrate the experimental paradigm. After 24h of treatment with drugs at 10μM, mRNAs from hES-derived MPCs were extracted and submitted to transcriptomic analysis to identify therapeutic regulation of genes involved in monogenic diseases, referenced in the OMIM database.

(B) Graphs show the number of drugs that regulate alternative splicing (LEFT) or transcript expression (RIGHT), categorized based on the number of events significantly altered compared to the control.

(C) Distribution of the different types of alternative splicing events induced by drug treatments.

(D) Graphs display the number of genes significantly regulated by each drug with log2 fold change >1 or ΔPSI >10% (blue) as well as the proportion of genes referenced in OMIM (red).

(E) Categorization of regulated genes referenced in OMIM by disease family.

(F) Normalized expressions of *EGR2, GDAP1, PMP22, LMNB1, SOD1, DES, PLP1,* and *SQSTM1* genes analyzed by quantitative RT-PCR in hES-derived MPCs treated with different drugs at 10μM for 24h. Data are shown as mean ± SD of three independent cultures and experiments.

Table 1. Drugs regulating disease-causing genes

Gene	Disease	OMIM number	Transmission	Molecular Genetic	Incidence	Drug Log2FC > 1.45	Regulation
PLP1	Pelizaeus-Merzbacher disease (PMD)	312080	recessive X linked	duplication	1/7663	rosuvastatin	Down
LMNB1	Adult-onset demyelinating leukodystrophy (ADLD)	169500	autosomic dominant	duplication	<1/1 000 000	rosuvastatin	Down
SQSTM1	Frontotemporal dementia/amyotrophic lateral sclerosis 3 (FTDALS type 3)	616437	autosomic dominant	loss of function	1-9/100 000	prazosin	UP
DES	Myofibrillar myopathy-1 (MFM1)	601419	autosomic dominant	loss of function	<1/1 000 000	manumycin rosuvastatin	UP

Log2FoldChange >1. The majority of compounds induced minimal transcriptional changes, with 72% (36 out of 50) altering the expression of fewer than 10 genes. DEGs analysis revealed that approximately 29% of the affected genes have previously been reported as mutated in monogenic diseases (Figures 1D and 1E; Table S2). RT-qPCR validation in mesodermal progenitors confirmed that eight such genes associated with pathogenic mutations were modulated by pentamidine, manumycin A, rosuvastatin, and prazosin (Figure 1F; Table 1). Notably, these three compounds were either not identified as regulators of these genes or were detected only in a limited number of cell lines in the Broad Institute's Connectivity Map database, suggesting cell-type-specific regulatory effects (Figure S2A). These findings were therefore further investigated in cell types relevant to the respective disease contexts. The impact of fludrocortisone, rosuvastatin and prazosin on the expression of genes associated with genodermatoses (*MMP1, ITGB4*), myopathy (*DES*), and FTD/ALS type 3 (*SQSTM1*) (Figure S2B) was validated by RT-qPCR in human keratinocytes, myoblasts, and hPSC-derived MNs (Figure S2C). These data demonstrate the potential of MPCs in identifying drug-gene regulatory interactions that can be conserved in other cell types than the mesenchymal lineage.

Validation of drug-gene interactions in patient-derived fibroblasts with rare monogenic diseases

The potential of these compounds to modulate target gene expression in a disease context was then assessed. However, due to the rarity of the associated conditions, access to patient-derived cells was limited. Fibroblasts were available only from patients with pathogenic mutations in *PLP1* and *SQSTM1* (Figure 2A).

PLP1 mutations are associated with Pelizaeus-Merzbacher disease (PMD), a rare degenerative central nervous system disorder (Cremers et al., 1987; W. H. Raskind et al., 1991). Fibroblasts carrying a *PLP1* gene duplication (Figure S3A), known to cause toxic overexpression and oligodendrocyte dysfunction (Readhead et al., 1994), were used in this study. RT-qPCR analysis confirmed its overexpression compared to unaffected fibroblasts (Figure 2B). The treatment of PMD fibroblasts with various doses of rosuvastatin for 24 h demonstrated that the drug effectively reduced PLP1 levels at both the RNA and protein levels in fibroblasts derived from patients with PMD (Figures 2B and 2C).

SQSTM1 is an autophagy receptor that recognizes and transfers ubiquitinated proteins for autophagic degradation. Mutations in this gene have been linked to FTD/ALS type 3 (Fecto et al., 2011). Fibroblasts harboring *SQSTM1* mutations (K238del and E396fs) were obtained from two patients (Figures S3B and S3C). Due to the loss of function caused by these heterozygous mutations, prazosin treatment was expected to enhance the cellular levels of functional SQSTM1 produced by the non-mutated allele. An increase in *SQSTM1* expression was confirmed in K238del and E396fs SQSTM1-mutated fibroblasts after 24 h of treatment with 10 μM of prazosin using RT-qPCR analysis (Figure 2D). Next, the impact of prazosin treatment on SQSTM1 protein levels was determined in both non-mutated and SQSTM1-mutated fibroblasts. Western blot analysis showed that treatment with 10 μM prazosin increased SQSTM1 levels in control and SQSTM1 K238del fibroblasts (Figures 2E and 2F). Increased levels of SQSTM1 after prazosin treatment were also validated by immunostaining in K238del and E396fs *SQSTM1*-mutated fibroblasts by quantifying the number of SQSTM1 puncta (Figures 2G and 2H), which corresponds to the SQSTM1 fraction associated with autophagosomes. Treatment with 10 μM of prazosin was found to be non-toxic to fibroblasts, as indicated by the cell count after treatment (Figure 2I). To assess whether the increase in SQSTM1 levels in response to prazosin resulted from autophagy inhibition, LC3B-II expression was analyzed by Western blot in fibroblasts treated with prazosin, the autophagy inhibitor bafilomycin A1, or their combination, with bafilomycin A1 added

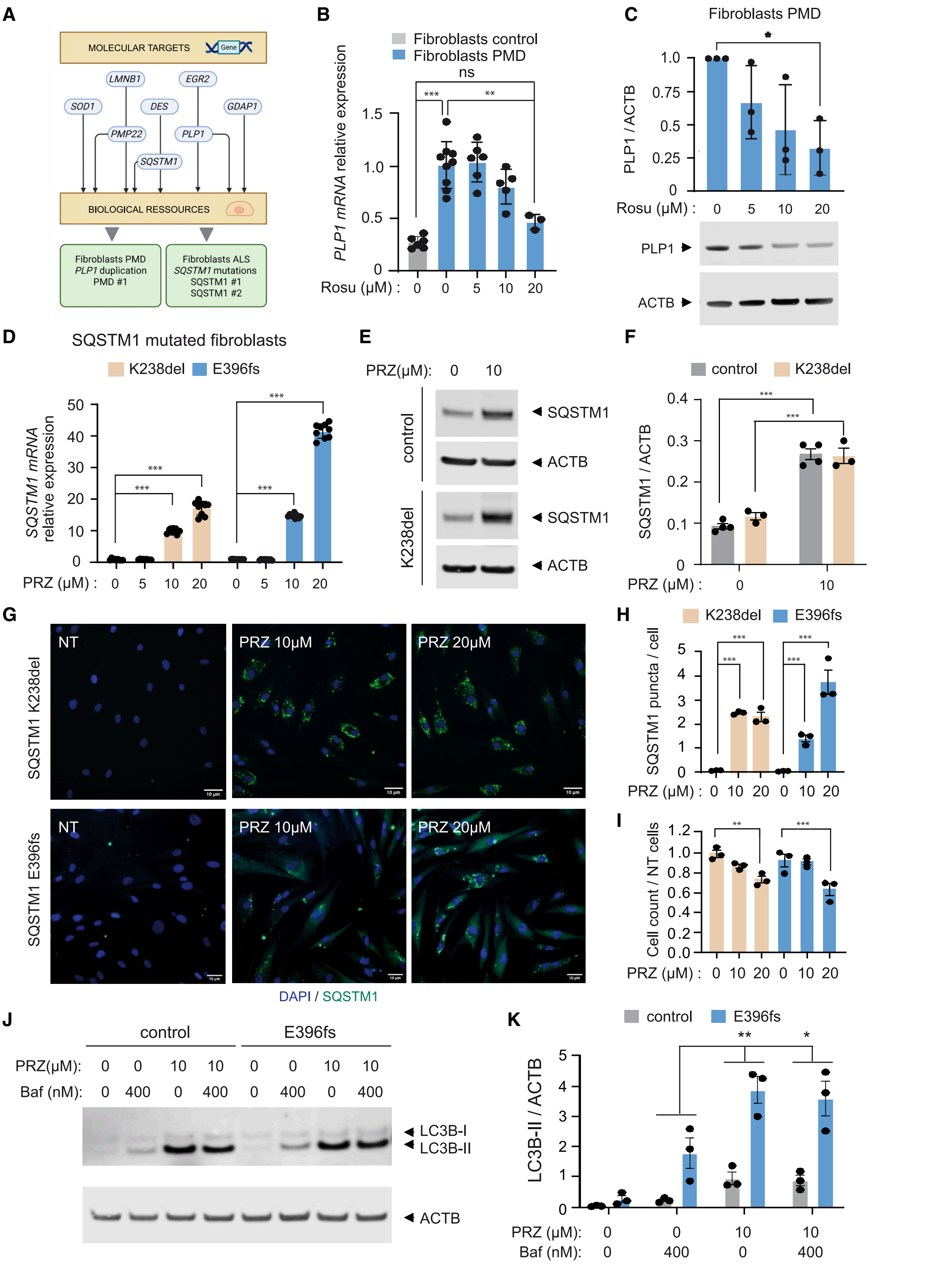

(legend on next page)

during the last 4 h of prazosin treatment. As expected, treatment with bafilomycin A1 alone, at a concentration of 400 nM, led to the accumulation of LC3B-II, reflecting impaired autophagic degradation. Notably, co-treatment with prazosin and bafilomycin A1 resulted in a further increase in LC3B-II levels compared to bafilomycin A1 treatment alone. These findings indicate that prazosin promotes SQSTM1 expression and enhances autophagosome formation, rather than inhibiting autophagy (Figures 2J and 2K).

Since SQSTM1 is known to play a key role in autophagy (Deng et al., 2020), a process central to ALS pathology, and prazosin has been shown to influence autophagy in other cellular models (An et al., 2019; Spencer et al., 2018), this compound was chosen for further investigation in the context of ALS.

Prazosin regulates several genes involved in autophagy

In addition to its impact on *SQSTM1*, the ability of prazosin to modulate other genes involved in autophagy was assessed. Gene set enrichment analysis (GSEA) of the prazosin transcriptomic signature in hES-derived MPCs revealed an upregulation of genes associated with autophagosomes, lysosomes, and endocytic vesicles (Figure 3A). Among these, seven genes (*MAP1LC3B, UBC, HSPA5, HSPB8, HSPA1A, HSPA1B,* and *HSPH1*) were identified as closely linked to *SQSTM1* using STRING software (Figure 3B). The increased expression of these seven genes was validated using RT-qPCR in non-mutated and *SQSTM1*-mutated (K238del and E396fs) fibroblasts treated with different doses of prazosin (Figure 3C). However, within the autophagic pathway, prazosin appears to selectively modulate specific genes. Notably, the expression of the autophagy receptor *OPTN*, involved in cargo recruitment to autophagosomes, remained unaffected by prazosin. This indicates that prazosin influences a subset of genes within the autophagy pathway.

Prazosin is an antihypertensive agent that lowers blood pressure by selective alpha-1-adrenergic receptor antagonism. Recently, terazosin, another alpha-adrenergic receptor antagonist, has been shown to improve MN phenotypes in several models of ALS by upregulating glycolysis and rescuing stress granule formation (Chaytow et al., 2022). We thus investigated whether terazosin and alfuzosin, an additional alpha-adrenergic receptor blocker, regulate *SQSTM1* expression. RT-qPCR analysis revealed that only prazosin could induce a pharmacologically dose-dependent regulation of the *SQSTM1* gene in both control and *SQSTM1* mutant fibroblasts (Figure 3D). These results suggest that prazosin induces specific cellular effects independently of the alpha-adrenergic receptor blockade it shares with other compounds of the same chemical family.

Figure 2. Rosuvastatin and prazosin regulate *PLP1* and *SQSTM1* transcripts in fibroblasts from PMD and FTD/ALS patients

(A) Schematic representation of the validation process for drug effects in fibroblasts derived from disease-affected patients.

(B) Graphs show *PLP1* transcript expression levels in PMD-fibroblasts, analyzed by RT-qPCR following treatment with varying doses of rosuvastatin, compared to non-affected fibroblasts. Data are presented as mean ± SEM from three independent cultures and experiments ($n = 3$). Statistical significance was determined using one-way ANOVA with Tukey's post hoc multiple comparisons test. $^{**}p \leq 0.01$ and $^{***}p \leq 0.001$.

(C) Western blotting analysis of PLP1 and ACTB proteins in PLP1 mutated fibroblasts treated with 5, 10, and 20μM of rosuvastatin. Data are shown as mean ± SEM of $n = 3$ independent cultures and experiments. Statistical significance was determined using one-way ANOVA with Dunnett's post hoc multiple comparisons test. $^{*}p \leq 0.05$.

(D) Graphs of *SQSTM1* transcript expression in FTD/ALS3 fibroblasts from two patients (SQSTM1 K238del and SQSTM1 E396fs), analyzed by RT-qPCR after 24 h treatment with various doses of prazosin. Data are represented as mean ± SEM from three independent cultures and experiments ($n = 3$). Statistical significance was determined using one-way ANOVA with Dunnett's post hoc multiple comparisons test. $^{***}p \leq 0.001$.

(E and F) Representative images (E) and quantification (F) of SQSTM1 analyzed by Western blot in wild-type and SQSTM1 K238del mutated fibroblasts treated with 10 μM prazosin for 24 h. Data are presented as mean ± SEM from more than three independent experiments ($n > 3$). Statistical significance was determined using unpaired Student's *t* test with Welch's correction. $^{***}p \leq 0.001$.

(G) Representative immunocytochemistry images showing DAPI staining and SQSTM1 staining in SQSTM1-mutated FTD/ALS fibroblasts (K238del and E396fs). Scale bars, 20 μm.

(H and I) Quantifications of nuclei and SQSTM1 puncta per cell following 24 h treatment with 10 or 20 μM prazosin, determined through automated microscope acquisition and analysis. Data are expressed as mean ± SD from three independent cultures and experiments ($n = 3$). Statistical significance was determined using one-way ANOVA with Dunnett's post hoc multiple comparisons test. $^{**}p \leq 0.01$ and $^{***}p \leq 0.001$.

(J and K) Representative images (J) and quantification (K) of LC3B analyzed by Western blot in non-mutated and SQSTM1 E396fs mutated fibroblasts treated with 10 μM prazosin for 24 h with and without 400 nM Bafilomycin A1 in the last 4 h. Data are presented as mean ± SEM from more than three independent experiments ($n > 3$). Statistical significance was determined using two-way ANOVA with Šídák's post hoc test. $^{*}p \leq 0.05$ and $^{**}p \leq 0.01$.

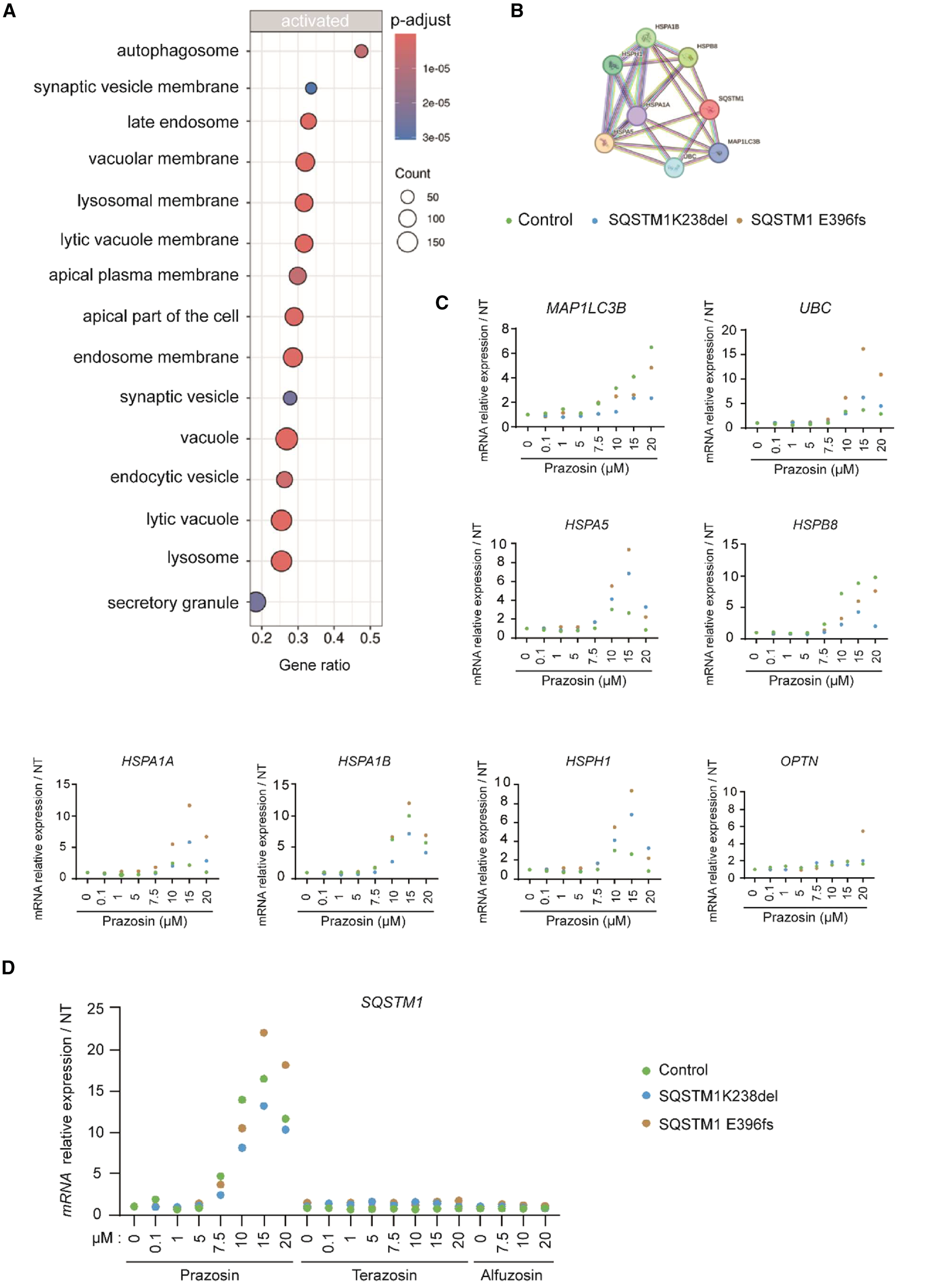

(legend on next page)

Prazosin induces SQSTM1 expression and autophagy in hES-derived motor neurons

Since ALS primarily affects MNs in the motor cortex, brainstem, and spinal cord, the impact of prazosin treatment on SQSTM1 expression in human MNs was evaluated. Spinal MNs were generated from hESCs using a previously developed protocol that produces over 75% ISLET1-positive (ISL1+) MNs within 14 days (Maury et al., 2015) (Figure 4A). A 24 h treatment with 10 μM prazosin led to increased *SQSTM1* transcript levels without affecting the number of ISLET1-positive cells (Figure S2C). This result was further confirmed at the protein level in MNs by an increased number of SQSTM1 puncta detected by immunostaining (Figures 4B and 4C). Prazosin treatment also increased LC3B-II levels, as observed by Western blot analysis (Figures 4D and 4E). Consistent with observations in fibroblasts, combined treatment with prazosin and bafilomycin A1 resulted in higher LC3B-II levels than treatment with bafilomycin A1 alone, confirming the induction of autophagosome formation in response to prazosin treatment (Figures 4D and 4E). These findings confirm that prazosin upregulates SQSTM1 and induces autophagy in hES-derived spinal MNs.

Modeling SQSTM1 loss of function in hiPSCs derived motor neurons

We next sought to evaluate the therapeutic potential of prazosin in a more pathological context of SQSTM1 loss of function. Using CRISPR-Cas9 technology, different hiPSC clones with SQSTM1 haploinsufficiency (*SQSTM1* +/−) or knockout (*SQSTM1* −/−) were generated (Figures S4A and S4B). The pluripotency of edited clones was validated by immunostaining for the markers OCT3/4, NANOG, and SSEA3, and their genomic integrity was confirmed by karyotypic analysis (Figures S4C and S4D). Potential off-target activity of sgRNAs was assessed by analyzing the most likely off-target sites identified by CRISPOR (Concordet and Haeussler, 2018) and no genome-editing-induced mutations were detected (Figure S5). Two hiPSC clones of each genotype were differentiated into spinal MNs, and SQSTM1 depletion was confirmed by western blot analysis (Figures 5A–5C).

As SQSTM1 is a key mediator of protein aggregate clearance, the impact of its depletion in MNs was first evaluated by assessing aggresome formation. Through its UBA and PB1 domains, SQSTM1 sequesters cytoplasmic protein aggregates and interacts with dynein to facilitate their transport to aggresomes, where they are subsequently degraded via autophagy. Proteostat staining was used to detect misfolded and aggregated proteins within aggresomes in *SQSTM1* +/+, +/−, and −/− MNs. A reduction in Proteostat signal was observed in SQSTM1-deficient MNs, reaching statistical significance in *SQSTM1* −/− MNs compared to controls (Figures 5D and 5E). Furthermore, treatment with the proteasome inhibitor bortezomib (BTZ), which promotes aggregate accumulation and subsequent clearance via autophagy, revealed a decrease in proteostat signal in *SQSTM1* +/− and −/− MNs relative to control MNs (Figures 5D and 5E). These findings highlight the critical role of SQSTM1 in mediating the trafficking of protein aggregates to aggresomes in MNs. The impact of SQSTM1 loss on autophagy was further evaluated by western blot analysis of autophagosome markers, through the quantification of LC3B-I and LC3B-II levels in *SQSTM1* +/+, +/−, and −/− hiPSC-derived MNs. Under basal conditions, LC3B-I and LC3B-II levels were comparable across genotypes. Upon autophagy induction with Torin-1, the increase in LC3B-II levels was reduced in *SQSTM1* +/− and −/− MNs compared to controls, indicating a diminished autophagic response in SQSTM1-deficient MNs (Figures 5F and 5G). At later stages of autophagy, Torin-1 treatment in control MNs resulted in a decrease in LAMP2 immunostaining area, an effect that was markedly diminished in *SQSTM1* +/− and −/− hiPSC-derived MNs (Figures S6A and S6B). Recent studies have reported defects in neuritic networks in ALS cellular models (Deng et al., 2020; Lefebvre-Omar et al., 2023; Mitsui et al., 2022). Immunostaining for TUJI, a member of the beta-tubulin protein family, confirmed that neurites in $SQSTM1^{+/-}$ and *SQSTM1*−/− hiPSC-derived MNs tended to retract into large bundles compared to control hiPSC-derived MNs. By day 24 of differentiation, the width of these neurites was significantly increased in SQSTM1-depleted MNs (Figures 5H and 5I). These observations suggest that decreased SQSTM1 levels in hiPSC-derived MNs impair autophagy and disrupt the structural integrity of the neurite network.

Figure 3. Prazosin regulates the expression of several genes involved in protein homeostasis and autophagy in fibroblasts from ALS3 patients

(A) Gene set enrichment analysis (GSEA) of transcriptomic data from hES-derived MPCs treated with 10 μM prazosin for 24 h. Data are analyzed using GSEA software to identify statistically significant enrichment in functionally related gene sets.

(B) STRING network analysis of genes regulated by prazosin treatment in hES-derived MPCs (log2 fold change >0.4) based on transcriptomic analysis after 24 h of treatment with 10 μM prazosin.

(C and D) Quantitative real-time PCR analysis of *MAP1LC3B, UBC, HSPA5, HSPB8, HSPA1A, HSPA1B, HSPH1, OPTN,* and *SQSTM1* gene expression was conducted in control fibroblasts and two ALS3 fibroblast lines treated for 24 h with varying doses of prazosin, terazosin, and alfuzosin.

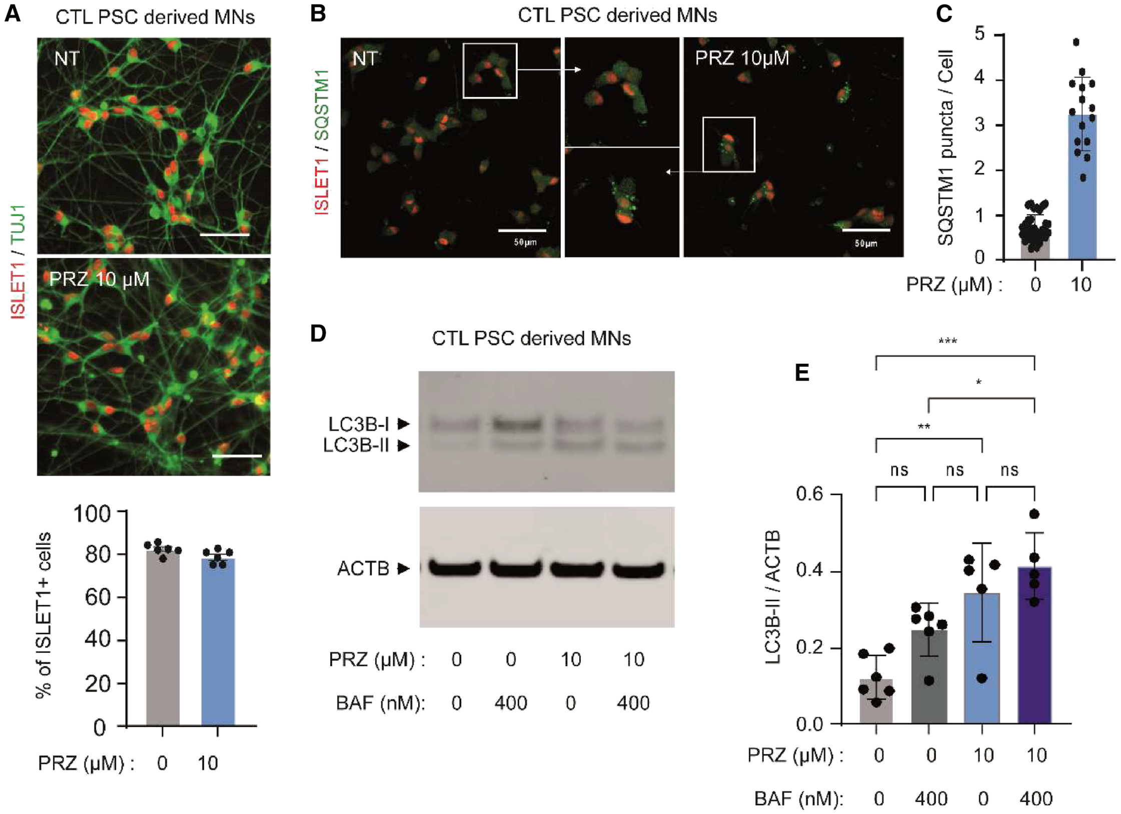

Figure 4. Prazosin induces SQSTM1 puncta and MAP1LC3B lipidation in hESC-derived motor neurons

(A) Representative immunocytochemistry images showing ISLET1 and TuJ1 staining in control hESCs-derived motor neurons (MNs), either untreated (NT) or treated with 10 μM prazosin for 24 h. The percentage of ISLET1+ cells is presented as a graph based on data obtained through automated image acquisition and analysis. Scale bars, 50 μm. Data are presented as mean ± SD from two independent differentiations from MN progenitors.

(B and C) Representative immunocytochemistry images showing ISLET1+ nuclei and SQSTM1 puncta staining in hESC-derived MNs after 24-h treatment with 10 μM prazosin. The number of SQSTM1 puncta per cell was quantified using automated microscope acquisition and analysis. Data are presented as mean ± SD from two independent differentiations from MN progenitors. Scale bars, 10 μm.

(D and E) Western blot analysis of MAP1LC3B modification and ACTB expression in control hiPSC-derived MNs treated with 10 μM prazosin for 24 h and bafilomycin (BAF) 400nM, 4 h before prazosin treatment ending. The displayed image represents one of the three independent differentiations from MN progenitors. Data are presented as mean ± SEM from three independent experiments. Statistical significance was determined using one-way ANOVA with Tukey's multiple comparisons post hoc test. $^{*}p \leq 0.05$, $^{**}p \leq 0.01$, and $^{***}p \leq 0.001$.

Prazosin treatment rescues autophagic and neuritic phenotypes in SQSTM1$^{+/-}$ hiPSC-derived motor neurons

Since prazosin treatment was found to increase *SQSTM1* transcript and protein levels without inhibiting autophagy in fibroblasts and hiPSC-derived MNs (Figure S2C; Figures 2E, 2F, 2J, and 2K; Figures 4D and 4E), its ability to restore the autophagic and neuritic phenotypes observed in MNs with SQSTM1 knockdown was evaluated. MNs derived from two *SQSTM1* +/+ and *SQSTM1* +/− hiPSC clones were treated with 10 μM prazosin or Torin-1 for 24 and 48 h. Western blot analysis revealed SQSTM1 upregulation at 24 and 48 h of treatment in response to prazosin only in *SQSTM1* +/+ MNs (Figure 6A). Despite a lower level of expression detected in *SQSTM1* +/− MNs, an increase in SQSTM1 protein levels was also observed in *SQSTM1* +/− MNs after 48 h of treatment with prazosin. When prazosin was combined with the autophagy inducer Torin-1, SQSTM1 levels were normalized, confirming the absence of autophagy blockade (Figure 6A). Immunostaining confirmed the organization of SQSTM1 into puncta following prazosin treatment in both *SQSTM1* +/+ and *SQSTM1* +/− MNs (Figure 6B). This was concomitant with the enhancement of LC3B-II levels in control and *SQSTM1* +/− MNs revealed by Western blot analysis (Figure 6C). Focusing on neuritic phenotypes, prazosin treatment significantly reduced neurite bundle formation in *SQSTM1* +/− MNs (Figures 6D and 6E) without affecting

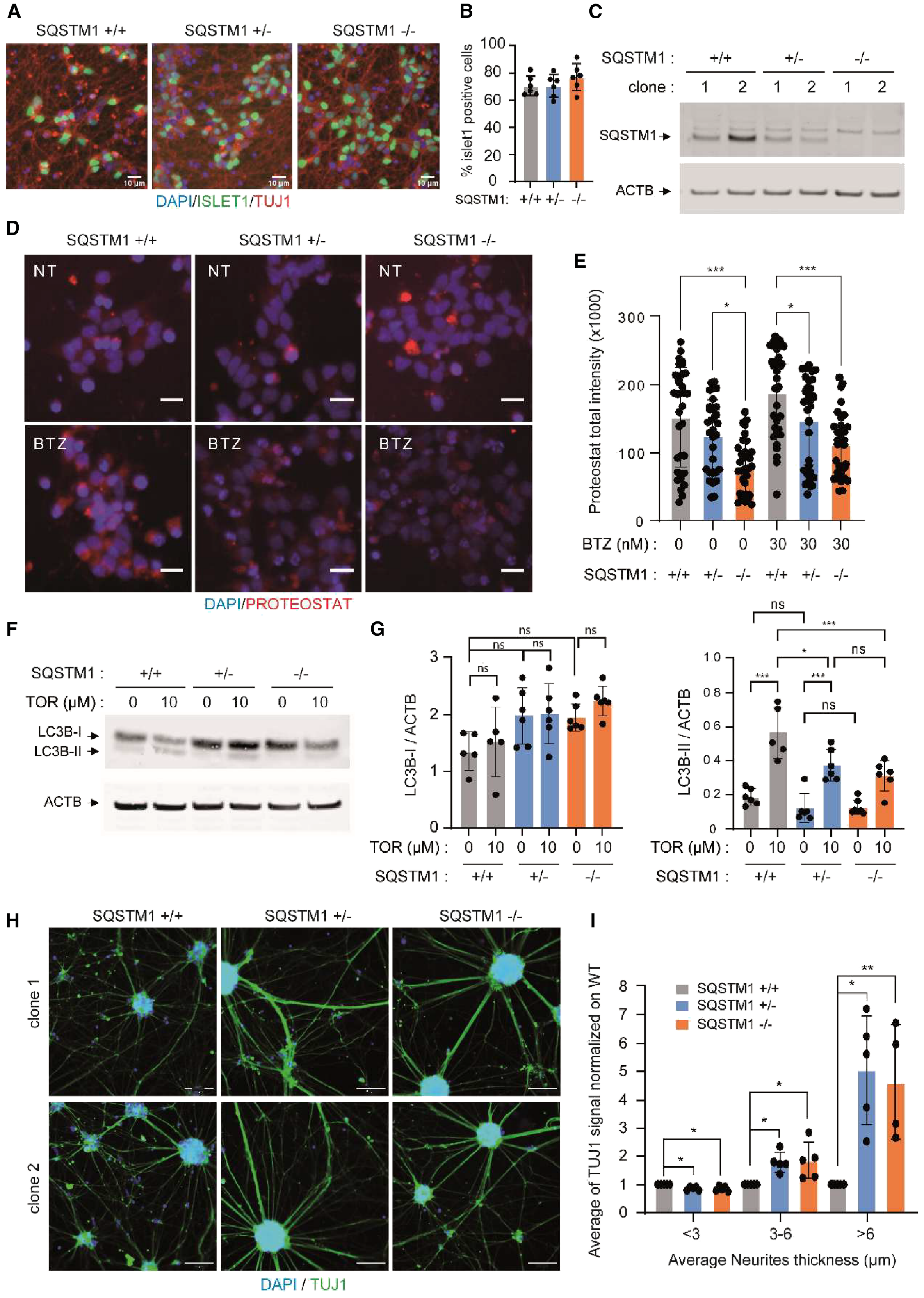

(legend on next page)

cell viability, as reflected by unchanged numbers of ISLET1 + MNs compared to controls (Figures 6F and 6G).

Prazosin improves the swimming of zebrafish with sqstm1 knockdown

To assess the therapeutic potential of targeting *SQSTM1* with prazosin *in vivo*, the drug was tested in a zebrafish model of ALS induced by SQSTM1 knockdown. As previously described (Lattante et al., 2015), wild-type zebrafish embryos injected with an antisense morpholino oligonucleotide (AMO) targeting the *sqstm1* ortholog exhibit shortened MN axons and swimming deficits, which were quantified using the touch-evoked escape response (TEER) test (Figures 7A and 7B). The absence of toxicity was first demonstrated after 4 days of treatment with prazosin in mismatch control embryos (Figure 7B). Subsequently, sqstm1-AMO-injected zebrafish embryos were treated with prazosin, resulting in a significant improvement in swimming behavior during the TEER test compared to untreated littermates (Figure 7B). To determine whether the beneficial effect of prazosin on zebrafish TEER responses was mediated by its α1-adrenergic receptor antagonistic activity, sqstm1-AMO-injected zebrafish embryos were treated with alfuzosin, an α1-adrenergic receptor antagonist that does not regulate SQSTM1 (Figure 3D). The lack of improvement in TEER scores following alfuzosin treatment indicates that the effects of prazosin are not due to α1-adrenergic receptor inhibition per se but are instead associated with increased SQSTM1 protein levels (Figure 7C; Figure S7).

DISCUSSION

This study proposes an alternative to the traditional drug screening paradigm by combining hPSC derivatives with deep RNA sequencing to map gene expression changes induced by pharmacological compounds. This strategy highlights a systematic framework for advancing the development of personalized therapies, particularly applicable to monogenic diseases for which DEGs, or an altered dosage of alternative transcripts, have been identified. In the present study, we explored, as proof of concept, the potential therapeutic effect of prazosin, an antihypertensive drug, as a modulator of SQSTM1 expression, a gene encoding the autophagy receptor SQSTM1/p62, implicated in rare forms of ALS (Fecto et al., 2011; Renton et al., 2014). Using patient fibroblasts, SQSTM1-depleted hiPSC-derived MNs, and a zebrafish sqstm1 knockdown model, prazosin was shown to enhance autophagic processes and restore MN phenotypes, including motor behavior *in vivo*.

Hypothesis-driven approaches have traditionally been used in drug repurposing, with cell-based assays used to explore overlaps between established pharmacological mechanisms and potential pathological pathways. The advent of disease-specific hPSCs has significantly advanced these methods (Rowe and Daley, 2019). In the present study, a paradigm shift is introduced using hPSCs in target-agnostic drug discovery approaches. Extending the scope of this research to a wider variety of hPSC-derived tissue types would be particularly beneficial in addressing the cell-type-dependent variability of pharmacological responses. Both our findings and the updated version of the Connectivity

Figure 5. Motor neurons with heterozygous or homozygous *SQSTM1* knockdown exhibit autophagy impairment and neuritic network perturbation

(A) Representative immunocytochemistry images show DAPI, ISLET1, and TUJ1 staining in hiPSC-derived MNs with *SQSTM1* +/+, *SQSTM1* +/−, and *SQSTM1* −/− genotypes after 14 days of differentiation. Scale bars, 10 μm.

(B) Quantification of the percentage of ISLET1+ MNs derived from control and *SQSTM1*-edited hiPSCs (two clones per genotype) using automated microscopic acquisition and analysis. Data are presented as mean ± SD from three independent differentiations of MN progenitors.

(C) Western blot analysis of SQSTM1 and ACTB expression in *SQSTM1* +/+, *SQSTM1* +/−, and *SQSTM1* −/− MNs derived from two edited hiPSC clones per genotype.

(D and E) Representative images and quantification of proteostat staining in *SQSTM1* +/+, *SQSTM1* +/−, and *SQSTM1* −/− MNs not treated (NT) or treated for 24 h with 30 nM bortezomib (BTZ). Scale bars, 20 μm Data are presented as mean ± SD from three independent differentiations from MN progenitors. Statistical significance was determined using one-way ANOVA with Tukey's multiple comparisons test. $^*p \leq 0.05$ and $^{***}p \leq 0.001$.

(F and G) Representative images and quantification of LC3B-II protein analyzed by Western blot after 24-h treatment with 10 μM Torin-1 in *SQSTM1* +/+, *SQSTM1* +/−, and *SQSTM1* −/− MNs derived from two individual edited hiPSC clones. Data are presented as mean ± SEM from more than three independent differentiations from MN progenitors. Statistical significance was determined using one-way ANOVA with Tukey's multiple comparisons test.

(H and I) Representative immunocytochemistry images of DAPI and TUJ1 staining in hiPSC-derived MNs with *SQSTM1* +/+, *SQSTM1* +/−, and *SQSTM1* −/− genotypes after 24 days of differentiation. Scale bars, 50 μm. TUJ1 staining in MNs with different *SQSTM1* genotypes was quantified and normalized to *SQSTM1* +/+ MNs across three neurite thickness intervals. Data are presented as mean ± SD from more than three independent differentiations from MN progenitors. Statistical significance was determined using the Kruskal-Wallis test with Dunnett's post hoc multiple comparisons test. ns: not significative, $^*p \leq 0.05$ and $^{**}p \leq 0.01$.

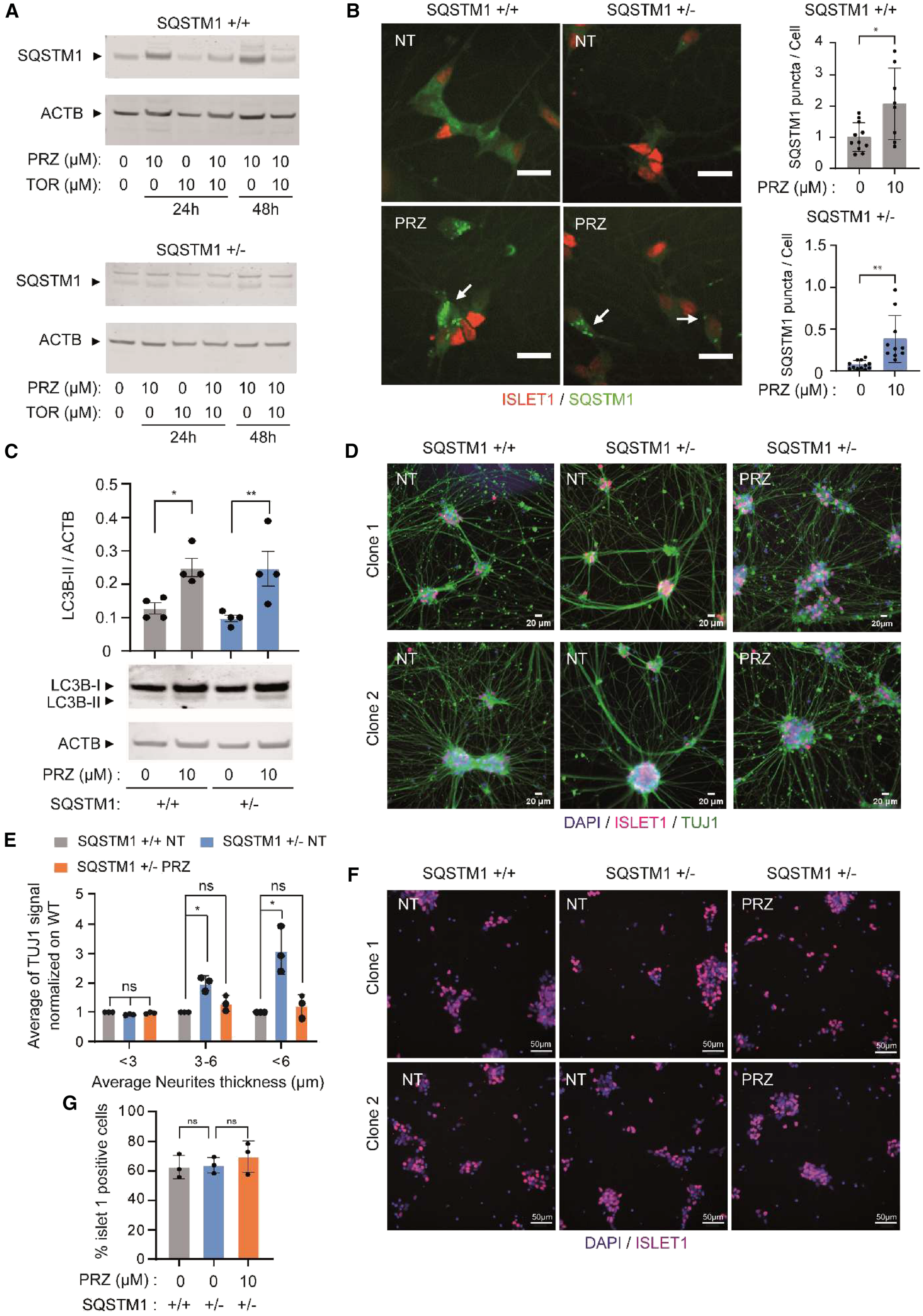

(legend on next page)

Map, which focuses on cancer cell lines, underline the importance of incorporating additional tissue types to better understand these responses (Subramanian et al., 2017). Our approach distinguishes itself from the Connectivity Map study by (1) using deep RNA sequencing, (2) analyzing both differential gene expression and splicing events, and (3) employing non-transformed cellular models. The ability of hPSCs to generate an unlimited supply of diverse cell types offers a powerful tool for exploring drug-specific gene expression profiles, potentially leading to the discovery of new therapeutic applications. Such a wide expansion would obviously benefit significantly from the integration of artificial intelligence (AI)-based methods to handle the vast amounts of data generated. Advances in AI are poised to address this challenge by enabling efficient data analysis and enhancing the scalability of these approaches (Sadybekov and Katritch, 2023). The combination of hPSC-derived cell diversity and AI-driven analytics promises to create a transformative platform for drug discovery, particularly for rare diseases for which the development of effective treatments has long been constrained by limited resources and inadequate biological models. This molecular screen was designed to detect changes in gene expression and splicing regulation. The proof-of-concept was performed using a single 10 μM dose, a concentration widely used in pharmacological screens, including the Broad Institute's Connectivity Map, allowing for direct comparison of overlapping compounds. In future studies, incorporating multiple doses and time points would broaden the scope and increase the resolution of the screening approach. As a preliminary proof of concept for the paradigm shift in drug discovery we propose, we have demonstrated the potential to repurpose prazosin for a rare form of ALS. Currently, there is no effective cure for ALS, and the few treatments available slow disease progression by only a few months. The heterogeneity of the disease, with numerous genetic mutations disrupting multiple molecular pathways, is one of the main challenges in developing a therapy for ALS. Over the past decades, research has highlighted the critical role of the autophagic pathway in ALS (Jagaraj et al., 2024). Defects at different stages of autophagy are evident in experimental models of familial ALS, involving genetic mutations such as *SOD1*, *SQSTM1*, *TDP-43*, and *OPTN*, but probably also play an important role in the pathogenesis of sporadic ALS (Chua et al., 2022). As a result, targeting the autophagic pathway has emerged as a potential therapeutic approach for ameliorating ALS-related pathological conditions, and several compounds that positively modulate autophagy, such as trehalose, rapamycin, and colchicine, are already in clinical trials (Castillo et al., 2013; Mandrioli et al., 2019, 2023). Our results confirm the therapeutic potential of autophagy induction for ALS and identify prazosin as a promising new approach.

Recently, terazosin, a structural analog of prazosin, was shown to improve MN phenotypes in several ALS models by activating the key glycolytic enzyme phosphoglycerate kinase 1 (Chaytow et al., 2022). Based on these results, a pilot clinical trial is currently underway to evaluate the potential of terazosin to reduce various biomarkers of neurodegeneration in patients with ALS. Nevertheless, our results demonstrated that neither terazosin nor alfuzosin induced a pharmacological increase in SQSTM1 expression. This suggests that prazosin regulates *SQSTM1* gene

Figure 6. Prazosin increases SQSTM1 levels in motor neurons with SQSTM1 haploinsufficiency and normalizes neurite network defects

(A) Representative images of SQSTM1 and ACTB expressions analyzed by Western blot in MNs derived from one control and *SQSTM1* +/− hiPSC clone, treated with 10 μM prazosin and 10 μM Torin-1 for 24 and 48 h.

(B) Representative images of immunocytochemistry for ISLET1 and SQSTM1 staining in MNs at 14 days of differentiation, derived from control and *SQSTM1* +/− hiPSCs, either untreated (NT) or treated with 10 μM prazosin (PRZ) for 24 h. Scale bars, 10μm. Data are presented as mean ± SD from two independent differentiations from MNs progenitors derived from 2 control and 2 *SQSTM1* +/− hiPSC clones ($n = 4$). Statistical significance was determined using Student's t test. $*p \leq 0.05$ and $**p \leq 0.01$.

(C) Representative images and quantification of LC3B-I, LC3B-II, and ACTB proteins analyzed by Western blot in MNs derived from two individual control and edited hiPSC clones, treated with 10 μM prazosin for 24 h. Data are presented as mean ± SEM from more than three independent differentiations from MN progenitors. Statistical significance was determined using one-way ANOVA with Dunnett's post hoc test. $*p \leq 0.05$ and $**p \leq 0.01$.

(D and E) Representative immunocytochemistry images (D) and quantification (E) of DAPI and TUJ1 staining in MNs at 24 days of differentiation derived from 2 *SQSTM1* +/+ and 2 *SQSTM1* +/− hiPSCs clones, either untreated (NT) or treated with 10 μM prazosin (PRZ) for 10 days. Scale bars, 10μm. TUJ1 staining in *SQSTM1* +/+ and *SQSTM1* +/− MNs was quantified and normalized to *SQSTM1* +/+ MNs across three neurite thickness intervals. Data are presented as mean ± SD from three independent differentiations from MNs progenitors from 2 clones per genotype. Statistical significance was determined using the Kruskal-Wallis test with Dunnett's post hoc multiple comparisons test. $*p \leq 0.05$.

(F and G) Representative immunocytochemistry images (F) and quantification (G) of DAPI and ISLET1 staining in MNs derived from 2 *SQSTM1* +/+ and 2 *SQSTM1* +/− hiPSCs clones at 18 days of differentiation, either untreated (NT) or treated with 10 μM prazosin (PRZ). Scale bars, 20 μm. Data are presented as mean ± SD from three independent differentiations from MN progenitors. Statistical significance was determined using a one-way ANOVA test. ns: not significant.

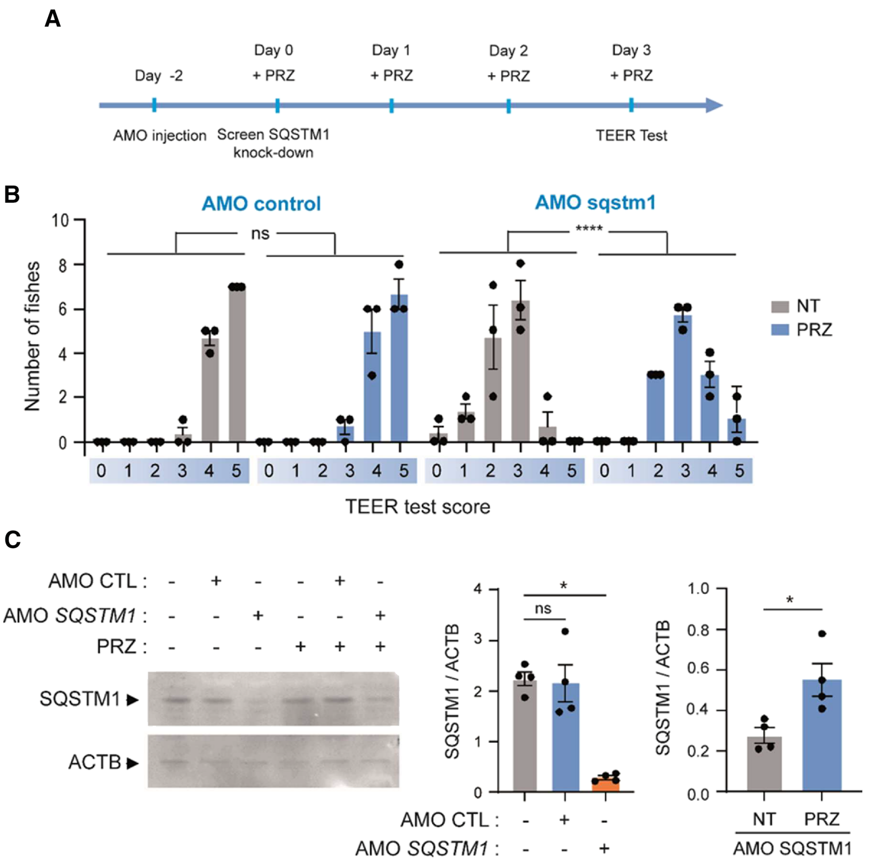

Figure 7. Prazosin alleviates the motor phenotype induced by sqstm1 knock-down in zebrafish

(A) Schematic illustration of zebrafish treatment and functional assessment.

(B) Quantification of swimming parameters in embryos injected with control or sqstm1-specific AMO, either untreated or treated with prazosin for 3 days, using a ViewPoint system. The qualitative scores ranged from 0 to 5. Data are presented as mean ± SEM from three independent experiments. Statistical significance was determined using the chi-square test for contingency data. $^{****}p \leq 0.0001$.

(C) Representative western blot images and corresponding quantification of SQSTM1 and ACTB expression in embryos injected with control or sqstm1-specific AMO, with or without prazosin treatment for 3 days. Data are presented as mean ± SEM from four technical replicates. Statistical significance was determined using one-way ANOVA with Dunnett's post hoc test and Student's *t* test. $^{*}p \leq 0.05$.

expression independently of alpha-adrenergic receptors. Recent studies support this hypothesis by showing that prazosin can enter cells via endocytosis, leading to the reorganization of the LAMP1-positive endo-lysosomal system (Fuchs et al., 2015; 2018). This is in accordance with the GSEA of prazosin-induced transcriptome, which highlights changes concerning the endolysosomal compartment. Fuchs et al. have demonstrated that prazosin is a lysosomotropic drug. It crosses membranes at neutral pH but becomes protonated and is impermeable to membranes at acidic pH. Lysosomal trapping of prazosin promotes lysosomal leakage, facilitating cross-presentation of antigens in dendritic cells (Fuchs et al., 2015). Lysosome damage is also known to trigger selective autophagy through the regulation of the TFE/MiTF bHLH transcription factor family, which are master regulators of lysosomal biogenesis and autophagy. This process involves MAP1LC3B lipidation (Nakamura et al., 2020) and the inhibition of TFEB phosphorylation by mTOR (Goodwin et al., 2021).

The effect of prazosin on autophagic processes appears, hence, to extend beyond the regulation of SQSTM1 expression. Our deep RNA sequencing data indicate that prazosin modulates the expression of other autophagy regulators, including proteins known to interact with SQSTM1, such as UBC and MAP1LC3B. The molecular mechanisms by which prazosin regulates the expression of these different autophagy players remain speculative at this stage. It is possible that prazosin acts on a common mechanism that controls the expression or stability of these different genes. A potential candidate for this common mechanism could be TFEB, a master transcriptional regulator of genes involved in lysosome biogenesis and autophagy (Sardiello et al., 2009). Our deep RNA sequencing data indicate that only a small proportion of TFEB direct targets belonging to the Coordinated Lysosomal Expression and Regulation (CLEAR) network were also regulated by prazosin. Interestingly, one member of the MiT-TFE family of basic helix-loop-helix leucine-zipper transcription factors, TFEC, was found to be robustly upregulated by prazosin in our RNA sequencing data. Further experiments examining nuclear/cytoplasmic redistribution are required to determine the implication of MiT-TFE family transcription factors in the molecular changes observed in response to prazosin treatment.

Our study has certain limitations. Firstly, the precise mechanisms by which prazosin influences autophagic

pathways remain unclear, requiring further experiments to elucidate its impact on transcriptional, post-transcriptional, and post-translational regulation. Secondly, although our results suggest that prazosin has therapeutic potential for ALS, its efficacy and safety need to be validated by further preclinical and clinical studies, particularly in various genetic contexts and in sporadic ALS. Given that prazosin has already been tested in numerous clinical trials for repositioning in conditions such as traumatic stress (Calohan et al., 2010; Cho et al., 2024; Mayer et al., 2023; M. A. Raskind et al., 2018) and Alzheimer's disease (L. Y. Wang et al., 2009), AI-driven analyses of data collected in these trials could provide valuable insights into its repositioning potential. Stratifying these data according to variables such as dosage and patient age could help uncover its true therapeutic potential. In addition, these approaches could also facilitate the selection of promising candidates for drug repurposing from the existing pharmacopeia. Finally, although hPSC-derived models offer unique opportunities to assess drug effects, replicating the complexity of *in vivo* environments and predicting patient-specific responses remain significant challenges. The inclusion of more complex hPSC-derived models, such as organoids, could offer new perspectives to overcome these limitations and develop this paradigm into a robust and reliable platform for drug discovery, not only for ALS but also for other complex and rare diseases.

In conclusion, our systematic approach to drug-specific gene expression profiling in hPSC derivatives lays the foundation for the development of more effective and personalized therapeutic strategies.

RESOURCE AVAILABILITY

Lead contact

Requests for further information and resources should be directed to the lead contact, Cécile Martinat (cmartinat@istem.fr).

Materials availability

All unique, stable reagents generated in this study are available from the lead contact upon reasonable request, contingent on the completion of a Materials Transfer Agreement.

Data and code availability

- Bulk RNA-seq data have been deposited at GEO: GSE261648 accession number and are publicly available as of the date of publication.
- Any additional information required to reanalyze the data reported in this paper is available from the lead contact upon request.

ACKNOWLEDGMENTS

We thank the France Génomique Platform for performing library preparation and sequencing. We gratefully acknowledge support from the PSMN (Pôle Scientifique de Modélisation Numérique) of the ENS de Lyon for the computing resources. The authors thank all the participants in the PULSE study and their families. The authors are grateful for financial support from the nonprofit research organization ARSLA (Christine Tabuenca, Marie-France Cazalère, Marie Léon, Valérie Goutines and Sabine Turgeman), and for support from the French clinical research networks FILSLAN and ACT4ALS-MND, and the Fédération de la Recherche Clinique du CHU de Lille (Prof David Devos, coordinator of the Pulse study, Anne-Sophie Rolland, Alain Duhamel, Maeva Kheng, Julien Labreuch, Dominique Deplanque, Edouard Millois, Victor Laugeais, Maxime Caillier, Aymen Aouni, Pauline Guyon, Francine Niset, Valérie Santraine, Marie Pleuvret, Mathilde Bon and Laetitia Thibault) to the PULSE study. We thank Laetitia Barrault, Benjamin Brinon, Raphaël Woelke, and Céline Buon (Team F Charbonnier, University Paris Cité & Inserm UMR_S1124, 75270, Paris Cedex 06, France) for their technical help. We express our gratitude to Pr Andrey Y. Abramov for providing SQSTM1-mutated fibroblasts. We express our gratitude to Pr Christine Baldeschi for providing adult normal keratinocytes. In memory of our friends and colleague Jacqueline Gide and Laetitia Barrault. Funding: Association Française contre les Myopathies (AFM-Téléthon), eRARE grant "eRECOGNITION" (ANR-18-RAR3-0007-02), Agence Nationale de la Recherche (ANR-10-LABX-73) and France Génomique. This section is to acknowledge contributions from non-authors and list funding sources. Because this section contains important information and many funding bodies require inclusion of grant numbers here, please check it carefully.

AUTHOR CONTRIBUTIONS

Conceptualization: S.B., M.P., and C.M.; methodology: S.B., M.C., A.B., C.B., E.K., M.P., and C.M.; validation: S.B. and C.M.; investigation: F.R., J.G, J.T., A.B., H.P., A.M., L.E.K., and S.B; resources: K. B., F.S., So.B., G.B., D.A., J.F.D., E.K., S.B., and J.M.P.; data curation: H.P.; D.A.; writing – original draft: S.B., M.P., and C.M.; writing, review & editing preparation: F.R., J.T., M.C., A.B., C.B., J.F.D., H.P., D. A., K.B., E.K., A.M., L.E., So.B., F.S., G.B., M.P., S.B., and C.M.; supervision: M.P. and C.M.; project administration: M.P., S.B., and C.M.; funding acquisition: C.M. and M.P.

DECLARATION OF INTERESTS

The authors declare no competing interests.

STAR★METHODS

Detailed methods are provided in the online version of this paper and include the following:

- KEY RESOURCES TABLE
- EXPERIMENTAL MODEL AND STUDY PARTICIPANT DETAILS
- METHOD DETAILS
  - Chemicals
  - Origin and culture of primary cells from patients
  - Differentiation, culture and treatment of hESC-derived MPCs

- ○ Generation of heterozygous and homozygous SQSTM1 knockout PSC lines
  - ○ Generation of spinal motoneurons from hiPSCs
  - ○ RNA sequencing library preparation, sequencing
  - ○ Bioinformatic analysis of splicing events and differential genes expression
  - ○ Gene expression analysis by quantitative RT-PCR
  - ○ Immunocytochemistry
  - ○ Aggresome Labeling
  - ○ Protein extraction and western blot analysis
  - ○ Prazosin treatment of zebrafish with sqstm1 knockdown
- QUANTIFICATION AND STATISTICAL ANALYSIS
- ADDITIONAL RESOURCES

SUPPLEMENTAL INFORMATION

Supplemental information can be found online at https://doi.org/10.1016/j.stemcr.2026.102977.

Received: November 25, 2025
Revised: May 19, 2026
Accepted: May 28, 2026
Published: June 25, 2026

REFERENCES

An, M., Ma, W.-H., Jia, H.-W., Li, Y.-W., Feng, J.-J., Meng, J., Qi, H., and Liu, J. (2019). Prazosin inhibits the growth and mobility of osteosarcoma cells. Transl. Cancer Res. *8*, 1997–2004. https://doi.org/10.21037/tcr.2019.09.03.

Anders, S., Pyl, P.T., and Huber, W. (2015). HTSeq—A Python framework to work with high-throughput sequencing data. Bioinformatics (Oxford, England) *31*, 166–169. https://doi.org/10.1093/bioinformatics/btu638.

Bartolome, F., Esteras, N., Martin-Requero, A., Boutoleau-Bretonniere, C., Vercelletto, M., Gabelle, A., Le Ber, I., Honda, T., Dinkova-Kostova, A.T., Hardy, J., et al. (2017). Pathogenic p62/SQSTM1 mutations impair energy metabolism through limitation of mitochondrial substrates. Sci. Rep. *7*, 1666. https://doi.org/10.1038/s41598-017-01678-4.

Benoit-Pilven, C., Marchet, C., Chautard, E., Lima, L., Lambert, M.-P., Sacomoto, G., Rey, A., Cologne, A., Terrone, S., Dulaurier, L., et al. (2018). Complementarity of assembly-first and mapping-first approaches for alternative splicing annotation and differential analysis from RNAseq data. Sci. Rep. *8*, 4307. https://doi.org/10.1038/s41598-018-21770-7.

Boissart, C., Poulet, A., Georges, P., Darville, H., Julita, E., Delorme, R., Bourgeron, T., Peschanski, M., and Benchoua, A. (2013). Differentiation from human pluripotent stem cells of cortical neurons of the superficial layers amenable to psychiatric disease modeling and high-throughput drug screening. Transl. Psychiatry *3*, e294. https://doi.org/10.1038/tp.2013.71.

Bolger, A.M., Lohse, M., and Usadel, B. (2014). Trimmomatic : A flexible trimmer for Illumina sequence data. Bioinformatics (Oxford, England) *30*, 2114–2120. https://doi.org/10.1093/bioinformatics/btu170.

Calohan, J., Peterson, K., Peskind, E.R., and Raskind, M.A. (2010). Prazosin treatment of trauma nightmares and sleep disturbance in soldiers deployed in Iraq. J. Trauma Stress *23*, 645–648. https://doi.org/10.1002/jts.20570.

Castillo, K., Nassif, M., Valenzuela, V., Rojas, F., Matus, S., Mercado, G., Court, F.A., van Zundert, B., and Hetz, C. (2013). Trehalose delays the progression of amyotrophic lateral sclerosis by enhancing autophagy in motoneurons. Autophagy *9*, 1308–1320. https://doi.org/10.4161/auto.25188.

Chaytow, H., Carroll, E., Gordon, D., Huang, Y.-T., van der Hoorn, D., Smith, H.L., Becker, T., Becker, C.G., Faller, K.M.E., Talbot, K., et al. (2022). Targeting phosphoglycerate kinase 1 with terazosin improves motor neuron phenotypes in multiple models of amyotrophic lateral sclerosis. EBioMedicine *83*, 104202. https://doi.org/10.1016/j.ebiom.2022.104202.

Cho, Y., Iliff, J.J., Lim, M.M., Raskind, M., and Peskind, E. (2024). A case of prazosin in treatment of rapid eye movement sleep behavior disorder. J. Clin. Sleep Med. *20*, 319–321. https://doi.org/10.5664/jcsm.10888.

Chua, J.P., De Calbiac, H., Kabashi, E., and Barmada, S.J. (2022). Autophagy and ALS : Mechanistic insights and therapeutic implications. Autophagy *18*, 254–282. https://doi.org/10.1080/15548627.2021.1926656.

Concordet, J.P., and Haeussler, M. (2018). CRISPOR: intuitive guide selection for CRISPR/Cas9 genome editing experiments and screens. Nucleic Acids Res. *46*, W242–W245. https://doi.org/10.1093/nar/gky354.

Cremers, F.P., Pfeiffer, R.A., van de Pol, T.J., Hofker, M.H., Kruse, T.A., Wieringa, B., and Ropers, H.H. (1987). An interstitial duplication of the X chromosome in a male allows physical fine mapping of probes from the Xq13-q22 region. Hum. Genet. *77*, 23–27. https://doi.org/10.1007/BF00284707.

Cummings, B.B., Marshall, J.L., Tukiainen, T., Lek, M., Donkervoort, S., Foley, A.R., Bolduc, V., Waddell, L.B., Sandaradura, S.A., O'Grady, G.L., et al. (2017). Improving genetic diagnosis in Mendelian disease with transcriptome sequencing. Sci. Transl. Med. *9*, eaal5209. https://doi.org/10.1126/scitranslmed.aal5209.

DeLuca, D.S., Levin, J.Z., Sivachenko, A., Fennell, T., Nazaire, M.-D., Williams, C., Reich, M., Winckler, W., and Getz, G. (2012). RNA-SeQC : RNA-seq metrics for quality control and process optimization. Bioinformatics (Oxford, England) *28*, 1530–1532. https://doi.org/10.1093/bioinformatics/bts196.

Deng, Z., Lim, J., Wang, Q., Purtell, K., Wu, S., Palomo, G.M., Tan, H., Manfredi, G., Zhao, Y., Peng, J., et al. (2020). ALS-FTLD-linked mutations of SQSTM1/p62 disrupt selective autophagy and NFE2L2/NRF2 anti-oxidative stress pathway. Autophagy *16*, 917–931. https://doi.org/10.1080/15548627.2019.1644076.

Dong, Z., Liu, Z., Liang, M., Pan, J., Lin, M., Lin, H., Luo, Y., Zhou, X., and Yao, W. (2021). Identification of circRNA-miRNA-mRNA networks contributes to explore underlying pathogenesis and therapy strategy of gastric cancer. J. Transl. Med. *19*, 226. https://doi.org/10.1186/s12967-021-02903-5.

Fecto, F., Yan, J., Vemula, S.P., Liu, E., Yang, Y., Chen, W., Zheng, J.G., Shi, Y., Siddique, N., Arrat, H., et al. (2011). SQSTM1 mutations

in familial and sporadic amyotrophic lateral sclerosis. Arch. Neurol. *68*, 1440–1446. https://doi.org/10.1001/archneurol.2011.250.

Fortney, K., Griesman, J., Kotlyar, M., Pastrello, C., Angeli, M., Sound-Tsao, M., and Jurisica, I. (2015). Prioritizing therapeutics for lung cancer: an integrative meta-analysis of cancer gene signatures and chemogenomic data. PLoS Comput. Biol. *11*, e1004068. https://doi.org/10.1371/journal.pcbi.1004068.

Fuchs, R., Stracke, A., Ebner, N., Zeller, C.W., Raninger, A.M., Schittmayer, M., Kueznik, T., Absenger-Novak, M., and Birner-Gruenberger, R. (2015). The cytotoxicity of the α1-adrenoceptor antagonist prazosin is linked to an endocytotic mechanism equivalent to transport-P. Toxicology *338*, 17–29. https://doi.org/10.1016/j.tox.2015.09.008.

Fuchs, R., Stracke, A., Holzmann, V., Luschin-Ebengreuth, G., Meier-Allard, N., Ebner, N., Lassacher, T.M., Absenger-Novak, M., Fröhlich, E., Schittmayer, M., et al. (2018). Prazosin induced lysosomal tubulation interferes with cytokinesis and the endocytic sorting of the tumour antigen CD98hc. Biochim. Biophys. Acta. Mol. Cell Res. *1865*, 1211–1229. https://doi.org/10.1016/j.bbamcr.2018.06.006.

Gonzalez-Fierro, A., and Duenas-Gonzalez, A. (2021). Drug repurposing for cancer therapy, easier said than done. Semin. Cancer Biol. *68*, 123–131. https://doi.org/10.1016/j.semcancer.2019.12.012.

Goodwin, J.M., Walkup, W.G., Hooper, K., Li, T., Kishi-Itakura, C., Ng, A., Lehmberg, T., Jha, A., Kommineni, S., Fletcher, K., et al. (2021). GABARAP sequesters the FLCN-FNIP tumor suppressor complex to couple autophagy with lysosomal biogenesis. Sci. Adv. *7*, eabj2485. https://doi.org/10.1126/sciadv.abj2485.

Jagaraj, C.J., Shadfar, S., Kashani, S.A., Saravanabavan, S., Farzana, F., and Atkin, J.D. (2024). Molecular hallmarks of ageing in amyotrophic lateral sclerosis. Cell. Mol. Life Sci. *81*, 111. https://doi.org/10.1007/s00018-024-05164-9.

Kim, D., Pertea, G., Trapnell, C., Pimentel, H., Kelley, R., and Salzberg, S.L. (2013). TopHat2 : Accurate alignment of transcriptomes in the presence of insertions, deletions and gene fusions. Genome Biol. *14*, R36. https://doi.org/10.1186/gb-2013-14-4-r36.

Lamb, J., Crawford, E.D., Peck, D., Modell, J.W., Blat, I.C., Wrobel, M.J., Lerner, J., Brunet, J.-P., Subramanian, A., Ross, K.N., et al. (2006). The Connectivity Map : Using gene-expression signatures to connect small molecules, genes, and disease. Science (New York, N.Y.) *313*, 1929–1935. https://doi.org/10.1126/science.1132939.

Lattante, S., de Calbiac, H., Le Ber, I., Brice, A., Ciura, S., and Kabashi, E. (2015). Sqstm1 knock-down causes a locomotor phenotype ameliorated by rapamycin in a zebrafish model of ALS/FTLD. Hum. Mol. Genet. *24*, 1682–1690. https://doi.org/10.1093/hmg/ddu580.

Lefebvre-Omar, C., Liu, E., Dalle, C., d'Incamps, B.L., Bigou, S., Daube, C., Karpf, L., Davenne, M., Robil, N., Jost Mousseau, C., et al. (2023). Neurofilament accumulations in amyotrophic lateral sclerosis patients' motor neurons impair axonal initial segment integrity. Cell. Mol. Life Sci. *80*, 150. https://doi.org/10.1007/s00018-023-04797-6.

Mandegar, M.A., Huebsch, N., Frolov, E.B., Shin, E., Truong, A., Olvera, M.P., Chan, A.H., Miyaoka, Y., Holmes, K., Spencer, C.I., et al. (2016). CRISPR Interference Efficiently Induces Specific and Reversible Gene Silencing in Human iPSCs. Cell Stem Cell *18*, 541–553. https://doi.org/10.1016/j.stem.2016.01.022.

Mandrioli, J., Crippa, V., Cereda, C., Bonetto, V., Zucchi, E., Gessani, A., Ceroni, M., Chio, A., D'Amico, R., Monsurrò, M.R., et al. (2019). Proteostasis and ALS : Protocol for a phase II, randomised, double-blind, placebo-controlled, multicentre clinical trial for colchicine in ALS (Co-ALS). BMJ Open *9*, e028486. https://doi.org/10.1136/bmjopen-2018-028486.

Mandrioli, J., D'Amico, R., Zucchi, E., De Biasi, S., Banchelli, F., Martinelli, I., Simonini, C., Lo Tartaro, D., Vicini, R., Fini, N., et al. (2023). Randomized, double-blind, placebo-controlled trial of rapamycin in amyotrophic lateral sclerosis. Nat. Commun. *14*, 4970. https://doi.org/10.1038/s41467-023-40734-8.

Maury, Y., Côme, J., Piskorowski, R.A., Salah-Mohellibi, N., Chevaleyre, V., Peschanski, M., Martinat, C., and Nedelec, S. (2015). Combinatorial analysis of developmental cues efficiently converts human pluripotent stem cells into multiple neuronal subtypes. Nat. Biotechnol. *33*, 89–96. https://doi.org/10.1038/nbt.3049.

Maury, Y., Poydenot, P., Brinon, B., Lesueur, L., Gide, J., Roquevière, S., Côme, J., Polvèche, H., Auboeuf, D., Alexandre Denis, J., et al. (2019). Pluripotent Stem Cell-Based Drug Screening Reveals Cardiac Glycosides as Modulators of Myotonic Dystrophy Type 1. iScience *11*, 258–271. https://doi.org/10.1016/j.isci.2018.12.019.

Mayer, C.L., Savage, P.J., Engle, C.K., Groh, S.S., Shofer, J.B., Hargrove, A.M., Williams, T.J., Poupore, E.L., Hart, K.L., Riechers, R. G., et al. (2023). Randomized controlled pilot trial of prazosin for prophylaxis of posttraumatic headaches in active-duty service members and veterans. Headache *63*, 751–762. https://doi.org/10.1111/head.14529.

Mitsui, S., Otomo, A., Sato, K., Ishiyama, M., Shimakura, K., Okada-Yamaguchi, C., Warabi, E., Yanagawa, T., Aoki, M., Shang, H.-F., and Hadano, S. (2022). SQSTM1, a protective factor of SOD1-linked motor neuron disease, regulates the accumulation and distribution of ubiquitinated protein aggregates in neuron. Neurochem. Int. *158*, 105364. https://doi.org/10.1016/j.neuint.2022.105364.

Nakamura, S., Shigeyama, S., Minami, S., Shima, T., Akayama, S., Matsuda, T., Esposito, A., Napolitano, G., Kuma, A., Namba-Hamano, T., et al. (2020). LC3 lipidation is essential for TFEB activation during the lysosomal damage response to kidney injury. Nat. Cell Biol. *22*, 1252–1263. https://doi.org/10.1038/s41556-020-00583-9.

Oana, K., Oma, Y., Suo, S., Takahashi, M.P., Nishino, I., Takeda, S., and Ishiura, S. (2013). Manumycin A corrects aberrant splicing of Clcn1 in myotonic dystrophy type 1 (DM1) mice. Sci. Rep. *3*, 2142. https://doi.org/10.1038/srep02142.

Oros, K.K., Arcand, S.L., Bayani, J., Squire, J.A., Mes-Masson, A.M., Tonin, P.N., and Greenwood, C.M.T. (2013). Analysis of genomic abnormalities in tumors: a review of available methods for Illumina two-color SNP genotyping and evaluation of performance. Cancer Genet. *206*, 103–115. https://doi.org/10.1016/j.cancergen.2013.03.001.

Parkesh, R., Childs-Disney, J.L., Nakamori, M., Kumar, A., Wang, E., Wang, T., Hoskins, J., Tran, T., Housman, D., Thornton, C.A., and Disney, M.D. (2012). Design of a bioactive small molecule that targets the myotonic dystrophy type 1 RNA via an RNA motif-ligand database and chemical similarity searching. J. Am. Chem. Soc. *134*, 4731–4742. https://doi.org/10.1021/ja210088v.

Raskind, M.A., Peskind, E.R., Chow, B., Harris, C., Davis-Karim, A., Holmes, H.A., Hart, K.L., McFall, M., Mellman, T.A., Reist, C., et al. (2018). Trial of Prazosin for Post-Traumatic Stress Disorder in Military Veterans. N. Engl. J. Med. *378*, 507–517. https://doi.org/10.1056/NEJMoa1507598.

Raskind, W.H., Williams, C.A., Hudson, L.D., and Bird, T.D. (1991). Complete deletion of the proteolipid protein gene (PLP) in a family with X-linked Pelizaeus-Merzbacher disease. Am. J. Hum. Genet. *49*, 1355–1360.

Readhead, C., Schneider, A., Griffiths, I., and Nave, K.A. (1994). Premature arrest of myelin formation in transgenic mice with increased proteolipid protein gene dosage. Neuron *12*, 583–595. https://doi.org/10.1016/0896-6273(94)90214-3.

Renton, A.E., Chiò, A., and Traynor, B.J. (2014). State of play in amyotrophic lateral sclerosis genetics. Nat. Neurosci. *17*, 17–23. https://doi.org/10.1038/nn.3584.

Roessler, H.I., Knoers, N.V.A.M., van Haelst, M.M., and van Haaften, G. (2021). Drug Repurposing for Rare Diseases. Trends Pharmacol. Sci. *42*, 255–267. https://doi.org/10.1016/j.tips.2021.01.003.

Rowe, R.G., and Daley, G.Q. (2019). Induced pluripotent stem cells in disease modelling and drug discovery. Nat. Rev. Genet. *20*, 377–388. https://doi.org/10.1038/s41576-019-0100-z.

Sadybekov, A.V., and Katritch, V. (2023). Computational approaches streamlining drug discovery. Nature *616*, 673–685. https://doi.org/10.1038/s41586-023-05905-z.

Sardiello, M., Palmieri, M., di Ronza, A., Medina, D.L., Valenza, M., Gennarino, V.A., Di Malta, C., Donaudy, F., Embrione, V., Polishchuk, R.S., et al. (2009). A gene network regulating lysosomal biogenesis and function. Science (New York, N.Y.) *325*, 473–477. https://doi.org/10.1126/science.1174447.

Sertkaya, A., Beleche, T., Jessup, A., and Sommers, B.D. (2024). Costs of Drug Development and Research and Development Intensity in the US, 2000-2018. JAMA Netw. Open *7*, e2415445. https://doi.org/10.1001/jamanetworkopen.2024.15445.

Spencer, B.H., McDermott, C.M., Chess-Williams, R., Christie, D., and Anoopkumar-Dukie, S. (2018). Prazosin but Not Tamsulosin Sensitises PC-3 and LNCaP Prostate Cancer Cells to Docetaxel. Pharmacology *102*, 10–18. https://doi.org/10.1159/000488713.

Subramanian, A., Narayan, R., Corsello, S.M., Peck, D.D., Natoli, T. E., Lu, X., Gould, J., Davis, J.F., Tubelli, A.A., Asiedu, J.K., et al. (2017). A Next Generation Connectivity Map : L1000 Platform and the First 1,000,000 Profiles. Cell *171*, 1437–1452.e17. https://doi.org/10.1016/j.cell.2017.10.049.

Vincent, F., Nueda, A., Lee, J., Schenone, M., Prunotto, M., and Mercola, M. (2022). Phenotypic drug discovery : Recent successes, lessons learned and new directions. Nat. Rev. Drug Discov. *21*, 899–914. https://doi.org/10.1038/s41573-022-00472-w.

Wang, L.Y., Shofer, J.B., Rohde, K., Hart, K.L., Hoff, D.J., McFall, Y. H., Raskind, M.A., and Peskind, E.R. (2009). Prazosin for the treatment of behavioral symptoms in patients with Alzheimer disease with agitation and aggression. Am. J. Geriatr. Psychiatry *17*, 744–751. https://doi.org/10.1097/JGP.0b013e3181ab8c61.

Wang, R., Helbig, I., Edmondson, A.C., Lin, L., and Xing, Y. (2023). Splicing defects in rare diseases : Transcriptomics and machine learning strategies towards genetic diagnosis. Brief. Bioinform. *24*, bbad284. https://doi.org/10.1093/bib/bbad284.

Zhao, Y., Chen, X., Chen, J., and Qi, X. (2023). Decoding Connectivity Map-based drug repurposing for oncotherapy. Brief. Bioinform. *24*, bbad142. https://doi.org/10.1093/bib/bbad142.

STAR★METHODS

KEY RESOURCES TABLE

REAGENT or RESOURCE	SOURCE	IDENTIFIER
**Antibodies**		
Mouse monoclonal anti-SQSTM1	Abcam	Cat# ab56416; RRID: AB_945626
Mouse monoclonal anti-LAMP2	Abcam	Cat# ab25631; RRID: AB_470709
Mouse monoclonal anti-Oct3/4	Santa Cruz	Cat# sc-5279; SantaCruz, RRID: AB_628051
Rabbit monoclonal anti-NANOG	Cell Signaling Technology	Cat# 4903; RRID: AB_10559205
SSEA3	BD Pharmingen	Cat# 560879; RRID: AB_10564070.
TUJ1/TUBB3	BioLegend	Cat# 801202; RRID: AB_2313773
Goat polyclonal anti-ISLET1	R and D Systems	Cat# AF1837; RRID: AB_2126324
Rabbit polyclonal anti-PLP	Abcam	Cat# ab28486; RRID: AB_776593
Rabbit polyclonal anti-LC3B	Novus	Cat# NB600-1384; RRID: AB_669581
Rabbit monoclonal anti-ACTB	LICORbio	Cat# 926-42210; RRID: AB_1850027
**Biological samples**		
Fibroblasts from patient carrying a *PLP1* duplication	Dr. Knut Brockmann Universitäts medizin Göttingen, Germany	N/A
Fibroblasts from patient carrying the K238 Del mutation in *SQSTM1* gene	Dr. Andrey Y. Abramov UCL Institute of Neurology Queen Square, UK	Bartolome et al., 2017
Fibroblasts from patient carrying the mutation c.1184_1187dupTTGA (p.Glu396fs) in the *SQSTM1* gene	Dr. François Salachas AP-HP, Paris ALS Centre, Hôpital de la Salpêtrière, Paris	N/A
**Chemicals, peptides, and recombinant proteins**		
Prazosin	Sigma-Aldrich	P7791
Alfuzosin	Sigma-Aldrich	A0232
Torin-1	Tocris	4247/10
Terazosin	Tocris	1506/50
Bortezomib	Selleckchem	S1013
Bafilomycin A	MedChemExpress	HY100558
**Critical commercial assays**		
Proteostat aggresome detection kit	ENZO Life Sciences	ENZ-51035-0025
**Deposited data**		
Raw and analyzed data	This paper	GEO: GSE261648
**Experimental models: Cell lines**		

(*Continued on next page*)

Continued

REAGENT or RESOURCE	SOURCE	IDENTIFIER
human Embryonic Stem Cell Line SA001	National Stem Cell Bank (NSCB), NIH Approved	Cellartis/Takara
human Induced Pluripotent Stem Cell Line 1432	IStem	N/A
human Induced Pluripotent Stem Cell Line 1869	IStem	N/A
human Induced Pluripotent Stem Cell Line 1869 SQSTM1 +/- clones 1	IStem	N/A
human Induced Pluripotent Stem Cell Line 1869 SQSTM1 +/- clones 2	IStem	N/A
human Induced Pluripotent Stem Cell Line 1869 SQSTM1 -/- clones 1	IStem	N/A
human Induced Pluripotent Stem Cell Line 1869 SQSTM1 -/- clones 2	IStem	N/A
**Experimental models: Organisms/strains**		
Zebrafish with SQSTM1 knock-down	Dr. Edor Kabashi, Laboratory of Translational Research for Neurological Disorders, Imagine Institute.	Lattante et al., 2015
**Oligonucleotides**		
Primers for Gene expression analysis, see Table S3	This paper	N/A
SQSTM1 +/- clones 1 sgRNA 109: AGCCATCGCAGATCACATTG GGG	This paper	SYNTHEGO
SQSTM1 +/- clones 2 sgRNA 116: CACCCCAATGTGATCTGCGA TGG	This paper	SYNTHEGO
SQSTM1 -/- clones 1&2 sgRNA 134: TCAGGAGGCGCCCCGCAACA TGG	This paper	SYNTHEGO
Primers for Off target analysis in SQSTM1 edited iPSC clones, see Table S3	This paper	N/A
AMO sqstm1-mis 5′-ATCAACAGACCGAAACTCTCATCCT-3′	Lattante et al., 2015	N/A
AMO sqstm1 5′-ATGAAGAGACGGAAAGTGTCATCCT-3′	Lattante et al., 2015	N/A
**Recombinant DNA**		
homology-directed repair (HDR) donor plasmid pAAVS1-PDi-CRISPRn	Mandegar et al., 2016	Addgene #73500
**Software and algorithms**		
GenomeStudio v2.0.5 software	Oros et al., 2013	Illumina
CRISPOR	Concordet and Haeussler, 2018	https://github.com/maximilianh/crisporWebsite/
Trimmomatic	Bolger et al., 2014	Trimmomatic/README.md at main · usadellab/Trimmomatic · GitHub
RNA-SeQC	DeLuca et al., 2012	www.broadinstitute.org/rna-seqc/
Tophat2	Kim et al., 2013	https://github.com/infphilo/tophat
Picard suite	Picard	http://www.broadinstittute.github.io/picard
TopHat2 (v2.0.853)	Kim et al., 2013	https://github.com/infphilo/tophat

(*Continued on next page*)

Continued

REAGENT or RESOURCE	SOURCE	IDENTIFIER
FaRLine (FasterDB RNAseq Pipeline)	Benoit-Pilven et al., 2018	https://kissplice.prabi.fr/pipeline_ks_farline/
DESeq2 R/ Bioconductor package (v1.10.1)	Anders et al., 2015	https://bioconductor.org/packages//release/bioc/html/DESeq2.html
GraphPad Prism version 11.0.0 for Windows	GraphPad Software, Boston, Massachusetts USA	www.graphpad.com

EXPERIMENTAL MODEL AND STUDY PARTICIPANT DETAILS

This study used the male human embryonic stem cell line SA001, for which IStem holds authorization from the French Biomedicine Agency (NOR: SSAB2133048S). The CRISPR/Cas9-edited male 1869 human induced pluripotent stem cell lines carrying the heterozygous or homozygous SQSTM1 gene knock-out were analysed alongside the isogenic wild-type parental line. In addition, the control male 1432 human induced pluripotent stem cell line was included.

Fibroblasts from patient with *PLP1* gene duplication or fibroblasts with mutations in the *SQSTM1* gene (K238 Del and p. Glu396fs) were respectively obtained from Dr. Knut Brockman, Dr. Andrey Abramov and Dr. François Salachas. Cells obtained from patients were collected after informed consent had been obtained. All protocols were approved by the Institutional Review Boards at the institutions involved. Mutations in the PLPL1 gene were confirmed by SNP analysis, while mutations in the SQSTM1 gene were validated using Sanger sequencing.

Larval zebrafish (*Danio rerio*) were maintained at the Imagine Institute (Paris) facility and bred according to the National and European Guidelines for Animal Welfare. Experiments were performed on wild-type zebrafish larvae from AB strains. Zebrafish were staged in terms of hours postfertilization (hpf) based on morphological criteria and manually dechorionated using fine forceps at 24 hpf. All the experiments were conducted on morphologically normal zebrafish larvae.

METHOD DETAILS

Chemicals

Small molecules listed in Table S1 and Bortezomib were purchased from Selleckchem. Prazosin (P7791) and alfuzosin (A0232) were purchased from Sigma-Aldrich®. Torin-1 (4247/10) and terazosin (1506/50) were purchased from Tocris. Bafilomycin A (HY100558) was purchased from MedChemExpress.

Origin and culture of primary cells from patients

All the primary cells obtained from patients were collected after informed consent had been obtained. All protocols were approved by the Institutional Review Boards at the institutions involved. Fibroblasts from a patient carrying a PLP1 duplication and healthy fibroblasts were obtained from Dr. Knut Brockmann (Universitätsmedizin Göttingen, Germany). PLP1 gene duplication was detected by SNP analysis. For that, high quality genomic DNA is obtained with QIAcube™ workstation using QIAamp® DNA Blood Mini Kit DNA Qiagen) from 5x106 cells. DNAg hybridation was achieved on Infinium Core-24v1-2 BeadChip (Illumina). Data were analyzed with GenomeStudio v2.0.5 software (Illumina) (Oros et al., 2013). Fibroblasts carrying the K238 Del mutation in SQSTM1 gene were provided by Dr. Andrey Y. Abramov (UCL Institute of Neurology Queen Square, UK) (Bartolome et al., 2017). Fibroblasts carrying the mutation c.1184_1187dupTTGA (p. Glu396fs) in the SQSTM1 gene were provided by Dr. François Salachas (AP-HP, Paris ALS Centre, Hôpital de la Salpêtrière, Paris). Fibroblasts were cultured in DMEM (Invitrogen) supplemented with GlutaMAX (Invitrogen), 10% foetal bovine serum (Sigma-Aldrich) and 1% non-essential amino acids (Invitrogen) on 0.1% gelatin coated flasks. All primary cells were tested every other week for the presence of mycoplasm using the MycoAlert Mycoplasma Detection Kit (Lonza).

Differentiation, culture and treatment of hESC-derived MPCs

MPCs were differentiated from SA001 hESCs as previously described (Maury et al., 2019). This hESC line was used following the recommendation of the French Law of Bioethics and declared at the French Agency of Biomedicine (Number SSAB2133048S). MPCs were cultured on 0.1% gelatin-coated flasks and plates (SigmaAldrich) using Knockout

Dulbecco's Modified Eagle's Medium (Invitrogen) supplemented with 20% fetal bovine serum (Eurobio, Les Ulis, France), 1mM Glutamax (Invitrogen), 1M non-essential amino acids (Invitrogen), and 0.1% β-mercaptoethanol (Invitrogen). For the RNA sequencing experiments, each compound was tested in triplicate at the dose of 10μM for 24 h. RNA were extracted from the cells using the RNeasy Micro Kit (Qiagen). The toxicity of the 50 compounds was assessed after 24h of treatment at 10 different doses using the CellTiter-Glo® Luminescent Cell Viability Assay (Promega).

Generation of heterozygous and homozygous SQSTM1 knockout PSC lines

To generate SQSTM1 depleted hiPSC clones, a control healthy hiPSC (1869) was engineered at the AAVS1 locus for doxycycline-inducible expression of SpCas9 protein using the homology-directed repair (HDR) donor plasmid pAAVS1-PDi-CRISPRn (Addgene #73500) (Mandegar et al., 2016). The generation and the use of this hiPSC line were approved by the french minister of health (2019-A02599-48). Briefly, hiPS cells were dissociated with StemPro Accutase Cell Dissociation Reagent (Gibco®), plated in 24-well plates at 25.000 cells per cm2 and treated with 50ng/mL doxycycline (Sigma-Aldrich®) to induce Cas9 expression. The next day, the cells were transfected with a mix of 10pmol of sgRNAs targeting SQSTM1 and tracrTNA using lipofectamine RNAiMax (Thermo Fisher Scientific) according to the manufacturer's protocol with appropriated guides targeting SQSTM1 and tracrRNA. sgRNAs targeting SQSTM1 sequence were determined by CRISPOR (Table S3). To select edited clones, genomic DNA was extracted from transfected hiPS cells either with QIAmp DNA Micro and Mini Kit (Qiagen) according to the manufacturer's instructions. Gene editing was analyzed by Restriction fragment length polymorphism (RFLP) with the BtgZI enzyme (Figure S4) and confirmed through Sanger sequencing. Genomic integrity was also verified by Multiplex fluorescence *in situ* hybridization (mFISH) karyotype analysis as described previously (Boissart et al., 2013). To determine off-target activity of our gRNAs, we analyzed by PCR the most likely off-target sites predicted by CRISPOR (http://crispor.tefor.net/). No mutation induced by genome editing was observed (Figure S4). The healthy hiPSC line 1432 was used as an additional control cell line. PSC lines were on vitronectin (ThermoFisher) coated dishes culture in StemMACS iPS-Brew XF medium (Miltenyi). Cells were passed manually or with EDTA for single cell dissociation and frozen in cryostore medium (Stemcell technologies). The identity of all PSC lines was verified by SNP analysis.

Generation of spinal motoneurons from hiPSCs

The conversion of control and SQSTM1 depleted hiPSC clones into spinal motoneurons was performed as previously described (Maury et al., 2015). Briefy, hiPSC were dissociated enzymatically using Stem Pro Accutase (ThermoFisher) and plated in 25 cm2 fasks (Dutscher) at $2x10^6$ cells per flask in an induced motoneuronal medium supplemented with cytokines every 2 days. After 10 days of differentiation, embryoid bodies were dissociated. Between days 10 and 14, MN progenitors are converted into MNs, and motoneuron phenotype was assessed by immunolabeling for ISLET1 (ISL1).

RNA sequencing library preparation, sequencing

Sequencing libraries were prepared using the llumina TruSeq Stranded mRNA Sample Prep Kit (Illumina, San Diego, CA). A 2 × 101 bp paired-end sequencing was performed on the HiSeq2000 instrument, using half a lane per sample, to produce on average 80 million read pairs per sample (160 million sequences) with an average insert length of 130 bp. The samples were sequenced at this depth to provide sufficient coverage for gene expression and alternative splicing analyses. Trimmomatic (Bolger et al., 2014), Tophat2 (Kim et al., 2013), Picard suite (http://www.broadinstittute.github.io/picard), RNA-SeQC (DeLuca et al., 2012) and in-house metrics were used to evaluate data quality. The quality control of the sequencing data was evaluated using FastQC. Reads were aligned using TopHat2 (v2.0.853). TopHat2 was run with the assistance of gene annotations (Illumina's iGenomes based on EnsEMBL r70), which means that the alignment was performed in three steps: transcriptome mapping, genome mapping, and spliced mapping.

Bioinformatic analysis of splicing events and differential genes expression

RNA-seq data from three biological replicates for each condition were analysed using FaRLine (FasterDB RNAseq Pipeline) in order to identify alternatively skipped exons (ASE), alternative 3′ splice sites (A3SS), alternative 5′ splice sites (A5SS), mutually exclusive exons (ME) and multiple exons skipping (Multi Skip) as previously described (Benoit-Pilven et al., 2018). A percent splicing index (PSI) value was calculated for each sample as the ratio of inclusion junction reads to the sum of inclusion and exclusion junction reads. As the datasets are paired, the difference in PSI values for each event (ΔPSI) was calculated as the median of ΔPSI values for each replicate. A filter is then applied on exon skipping events detected to select significant variants with an adjusted P value $\leq$0.05 and a ΔPSI value $\geq$10%. The gene expression level in

each sample was calculated with HTSeq-count (v0.8.0) and differential gene expression between conditions was computed with DESeq2 R/ Bioconductor package (v1.10.1) (abs(log2FoldChange) $\geq$ 0.4 and 1.45, p $\leq$ 0.05) (Anders et al., 2015). The dataset supporting the conclusions of this article is available in the GEO database repository (GEO: GSE261648).

Gene expression analysis by quantitative RT-PCR

Total RNA was extracted using the RNeasy Micro/Mini kit (Qiagen) and reverse transcribed using random hexamers and Superscript III Reverse Transcriptase kit (Invitrogen®) according to the manufacturer's protocol. Quantitative PCR reactions were carried out in 384-well plates using a QuantStudio 12K Flex Real-Time PCR System (Applied Biosystems) with Power SYBR Green 2× Master Mix (Life Technologies®), 0.5 µl of cDNA, and 100 nmol/l of primers (Invitrogen®) in a final volume of 10 µl. Detailed information on the primers sequence is provided in Table S3. Data were expressed as mean ± SD.

Immunocytochemistry

After fixation with 4% paraformaldehyde (Euromedex) for 15 minutes at room temperature, cells were incubated overnight at 4°C with the following primary antibodies : SQSTM1 (ab56416, ABCAM, RRID: AB_945626), LAMP2 (ab25631, ABCAM, RRID: AB_470709), OCT3/4 (sc-5279 c-10, SantaCruz, RRID : AB_628051), NANOG (D73G4, Cell Signalling, RRID : AB_10559205), SSEA3 (5600308, BD Pharmingen, RRID: AB_10564070), ISLET1 (AF1837, R&D Systems, RRID: AB_2126324) and TUJ1 (PRB-435P, BioLegend, RRID: AB_2313773). Appropriated secondary antibodies conjugated to Alexa fluorophores (Thermo Fisher Scientific) together with Hoechst 33258 (5 µg/ml; Sigma-Aldrich®) were next applied to the cells. Images were acquired on a Zeiss inverted fluorescence microscope with the Zen blue software (Zeiss®) or with the HCS CellInsight CX7 device (Thermo Fisher).

Aggresome Labeling

Human iPSC derived MNs were fixed with 4%paraformaldehyde for 10 minutes at room temperature. Cells were washed two times with PBS and permeabilized with 0.5% Triton X-100, 3 mM EDTA, pH 8.0, in 1× assay buffer of the Proteostat aggresome detection kit (ENZO Life Sciences). After two washes with PBS, 1× assay buffer supplemented with Proteostat detection reagent (1/1000) and Hoechst 33342 (500 ng/mL) was added to the cells for 30 minutes at room temperature. Cells were washed two times with PBS before imaging with the HCS CellInsight CX7 device (Thermo Fisher).

Protein extraction and western blot analysis

Cells were lysed in RIPA buffer (Sigma-Aldrich) containing 1% protease inhibitors (Sigma-Aldrich) and 10% phosphatase inhibitors (Roche). Proteins were quantified by BCA Protein Assay kit (Pierce). Protein extracts (10 to 20µg) were loaded on 4–12% Nu-PAGE Bis-Tris gels (Invitrogen) under reducing conditions and transferred to nitrocellulose membranes (Invitrogen) using the iBlot2 Dry Blotting System (Invitrogen). For LC3B protein analysis, PVDF Gel Transfer Stacks membranes were used (Invitrogen). Membranes were next incubated with Blocking buffer (LI-COR) supplemented with 0.1% Tween-20 and incubated overnight at 4°C with primary antibodies. Membranes were then incubated for 1 hour with the corresponding IRDye secondary antibodies (LI-COR) and immunoreactive protein bands were detected using an Odissey CLx Imager (LI-COR) according to the manufacturer's protocol. Antibodies used in Western blotting analysis were anti-PLP1 antibody (ab28486, ABCAM, RRID: AB_776593), anti-SQSTM1 antibody (ab56416, ABCAM, RRID: AB_945626), anti-LC3B antibody (NB600-1384, Novus, RRID: AB_3750600) and anti-ACTB antibody (92642210, LI-COR, RRID: AB_1850027).

Prazosin treatment of zebrafish with sqstm1 knockdown

Zebrafish were microinjected with Morpholino antisense oligonucleotides (AMO) complementary to the zebrafish SQSTM1 orthologue, sqstm1 (5′-ATGAAGAGACGGAAAGTGTCATCCT-3′) or with a control AMO (sqstm1-mis) (5′-ATCAACAGACCGAAACTCTCATCCT-3′), containing five mismatch nucleotides as previously described (Lattante et al., 2015). 48 hpf embryos were submitted to Touch-evoked escape response test (TEER) prior to drug treatment and were incubated overnight in 96-well plates. Prazosin and alfuzosin were added to the embryo's water at the final concentration of 10 µM. Spontaneous swimming between 6 and 48 h after the treatment was captured using a Zebralab system (ViewPoint, France). Embryos were recorded always at the same time. The scoring performed was qualitative within the multi-well plates following treatment. The scoring ranged from 0-5 with 0-representing dead; 1-alive but moving very little; 2-very short and slow bouts of swimming; 3-short bouts of swimming; 4-relatively long and fast bout of swimming

but for a shorter duration; 5-swimming like non-injected and non-treated controls. SQSTM1 expression level in zebrafish was analysis by electrophoresis and western blot using SQSTM1 (ab56416, ABCAM, RRID: AB_945626) and ACTB (92642210, LI-COR, RRID: AB_1850027) antibodies.

QUANTIFICATION AND STATISTICAL ANALYSIS

Data was plotted using GraphPad prism (version 10.2.0) and presented as the mean ± SD or mean± SEM for western blot analysis. Statistical analysis was performed using Student's t-test to compare the significative difference between 2 groups (Figures 2F, 6B, and 7C). To compare more than 2 groups, we used a parametric ANOVA test when data number > 30 (Figure 6G). If the ANOVA test was significant, a post-hoc test was performed. We used a Tukey's multiple comparisons test to determine the significative difference of one group with other groups (Figures 2B, 4D, 5E, and 5G) while a Dunnett's post hoc multiple comparisons test was performed to compare one group with the control group (Figures 2C, 2D, 2I, 6C, and 7C). To compare more than 2 groups when data number was <30, we used a non-parametric Kruskal-Wallis's test followed with Dunnett's post hoc multiple comparisons test to compare one group with the control group (Figures 5I and 6E). To determine the effect of drug treatment upon the fibroblast's genotype, a two-way ANOVA with Šídák's post hoc test was used in Figure 2K; Figure S6. To determine the effect of drug treatment in *sqstm1* CTL and *sqstm1-* zebrafishes, a Chi-square test for contingency data test was used in Figure 7B; Figure S7. "n" represents the number of independent experiments. Asterisks indicate significance level * $p \leq 0.05$, ** $p \leq 0.01$, *** $p \leq 0.001$, **** $p \leq 0.0001$. Differences with p values < 0.05 were regarded as significant.

ADDITIONAL RESOURCES

There are no additional resources.

Stem Cell Reports, Volume *21*

Supplemental Information

Integrative analysis of drug-gene signatures in human pluripotent stem cells reveals prazosin as a novel SQSTM1 regulator for ALS therapeutics

Florine Roussange, Jacqueline Gide, Johana Tournois, Michel Cailleret, Anne Boland, Christophe Battail, Jean-François Deleuze, Hélène Polvèche, Didier Auboeuf, Knut Brockmann, Edor Kabashi, Anca Marian, Lina El Kassar, Sophie Blondel, François Salachas, Gaëlle Bruneteau, Marc Peschanski, Cécile Martinat, and Sandrine Baghdoyan

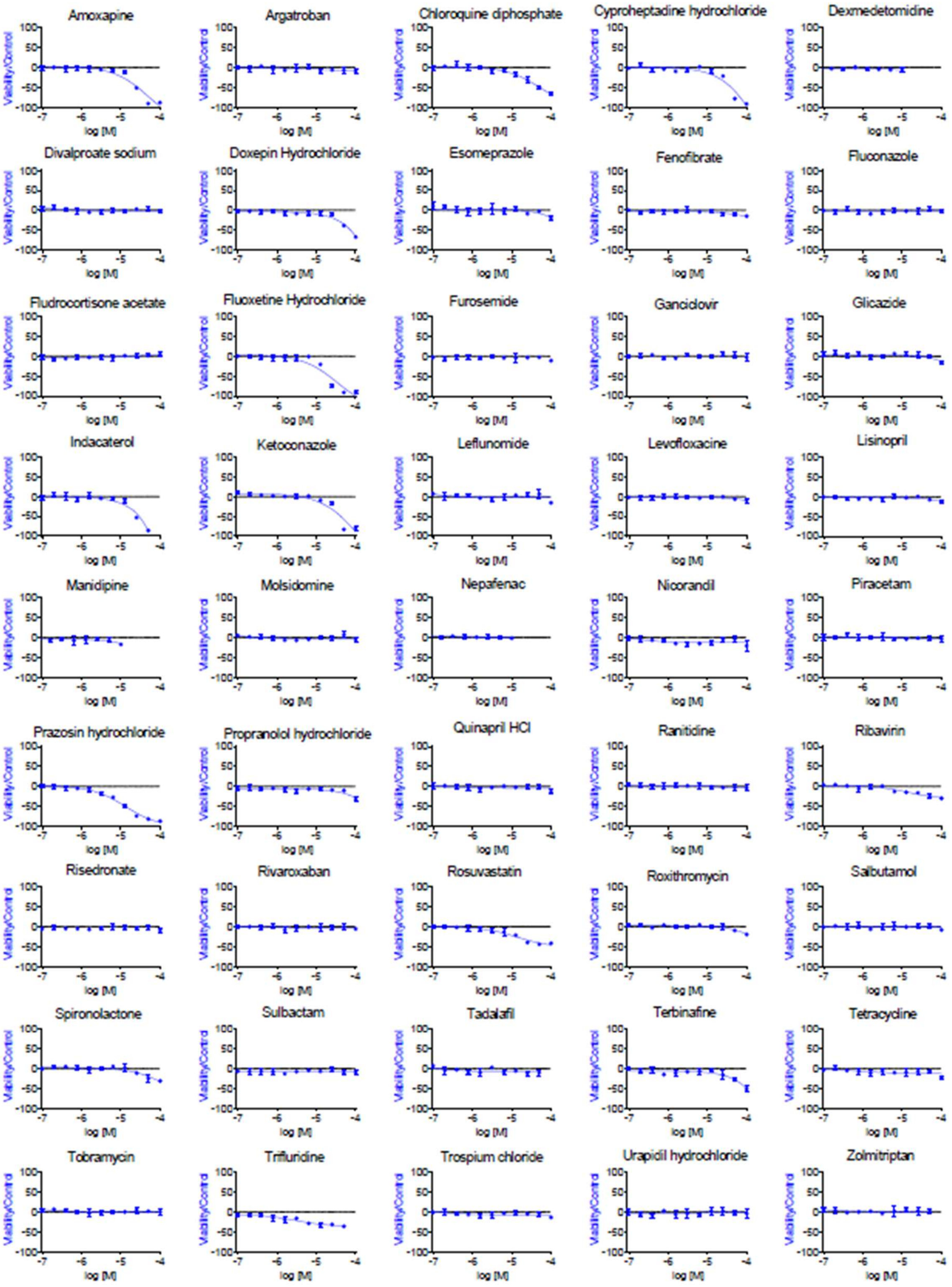
Amoxapine
Argatroban
Chloroquine diphosphate
Cyproheptadine hydrochloride
Dexmedetomidine
Divalproate sodium
Doxepin Hydrochloride
Esomeprazole
Fenofibrate
Fluconazole
Fludrocortisone acetate
Fluoxetine Hydrochloride
Furosemide
Ganciclovir
Glicazide
Indacaterol
Ketoconazole
Leflunomide
Levofloxacine
Lisinopril
Manidipine
Molsidomine
Nepafenac
Nicorandil
Piracetam
Prazosin hydrochloride
Propranolol hydrochloride
Quinapril HCl
Ranitidine
Ribavirin
Risedronate
Rivaroxaban
Rosuvastatin
Roxithromycin
Salbutamol
Spironolactone
Sulbactam
Tadalafil
Terbinafine
Tetracycline
Tobramycin
Trifluridine
Trospium chloride
Urapidil hydrochloride
Zolmitriptan
Viability/Control
log [M]

Figure S1. **Viability of hES-derived MPCs treated with the compounds of the smart repositioning library, related to Figure 1.** Cell viability of hES-derived MPCs was assessed using the Cell Titer-Glo assay following 24-hour treatment with various doses of drugs from the repositioning library.

A

		Nb of cell line with 2 < DE Z score > 2 / nb total of cell lines
Manumycin A	*LMNB1*	6/47
	SOD1	2/47
Rosuvastatin	*DESMIN*	2/13
	LMNB1	1/13
	PLP1	0/13
Prazosin	*PMP22*	0/13
	SQSTM1	4/13
Pentamidine	*EGR2*	non analyzed
	GDAP1	non analyzed
	PMP22	non analyzed

B

Gene	Mutation	Disease (OMIM)	Reference	Drug	DE FC in hES derived MSCs
MMP1	AR	RDEB (226600)	Titeux et al., 2008	Fludrocortisone acetate	-0,94
ITGB4	AR	JEB5A (619816)	Inoue et al., 2000	Rosuvastatin	3,28
DES	AD	MFM1 (601419)	Munoz-Marmol et al., 1998	Rosuvastatin	1,91
SQSTM1	AD	FTDALS3 (616437)	Fecto et al., 2011	Prazosin	2.43

C

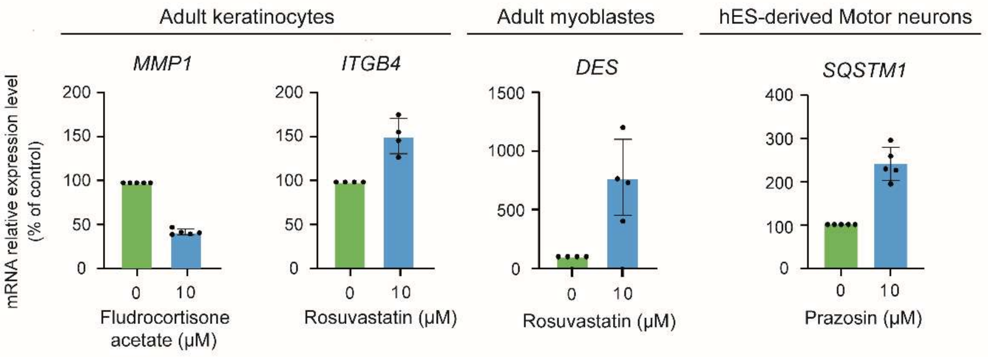

Figure S2. Analysis of gene expression regulation in various cellular contexts, related to Figure 1.

(A) Gene regulations validated by RT-qPCR in hES-derived MPCs were analyzed using the Broad Institute's Connectivity Map database.

(B) Identification of 4 genes - *MMP1* (matrix metallopeptidase 1), *ITGBA* (integrin subunit beta 4), *DES* (Desmin) and *SQSTM1* (Sequestosome 1) associated with a monogenic disease and responsive to treatment with drugs from the smart drug library.

(C) Plots of relative transcript expression for *MMP1, ITGB4, DES* and *SQSTM1* in human adult keratinocytes, human adult myoblasts and hES derived motor neurons (MNs) analyzed by RT-qPCR following 24-hour treatment with 10 µM of fludrocortisone acetate, rosuvastatin and prazosin. Data are presented as mean ± SD from three independent experiments.

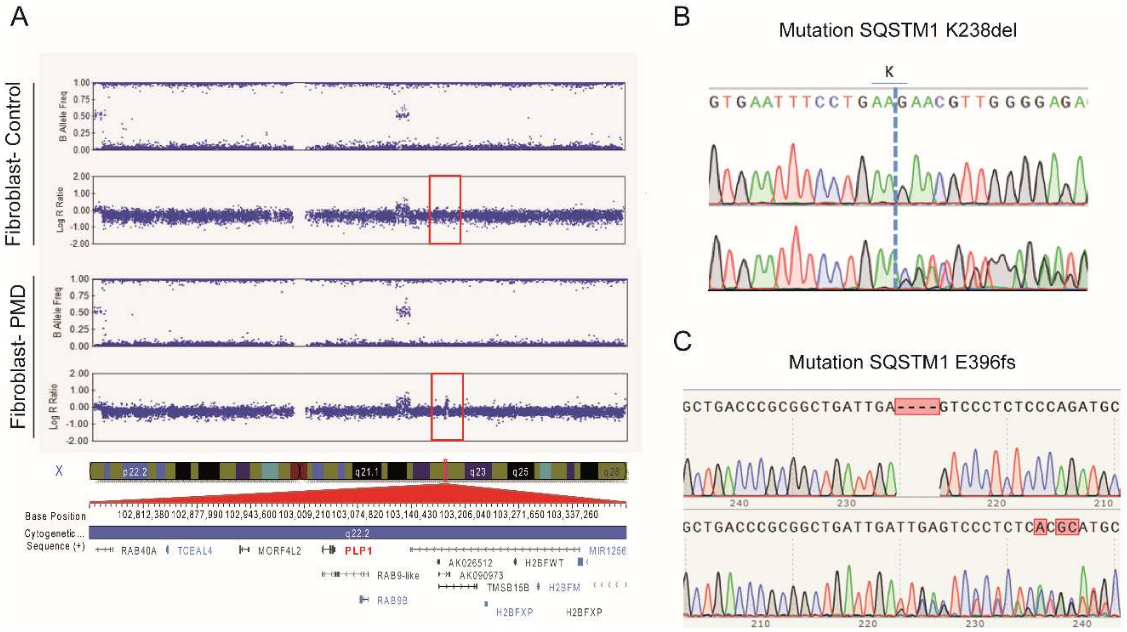

Figure S3. Identification of genetic alterations in pathological fibroblasts, related to Figure 2.

(A) SNP analysis of chromosome X in fibroblasts from non-affected or PMD affected patients.

(B and C) Sanger sequencing analysis of mutations in SQSTM1 K238del and SQSTM1 E396fs fibroblasts of FTD/ALS3 patients.

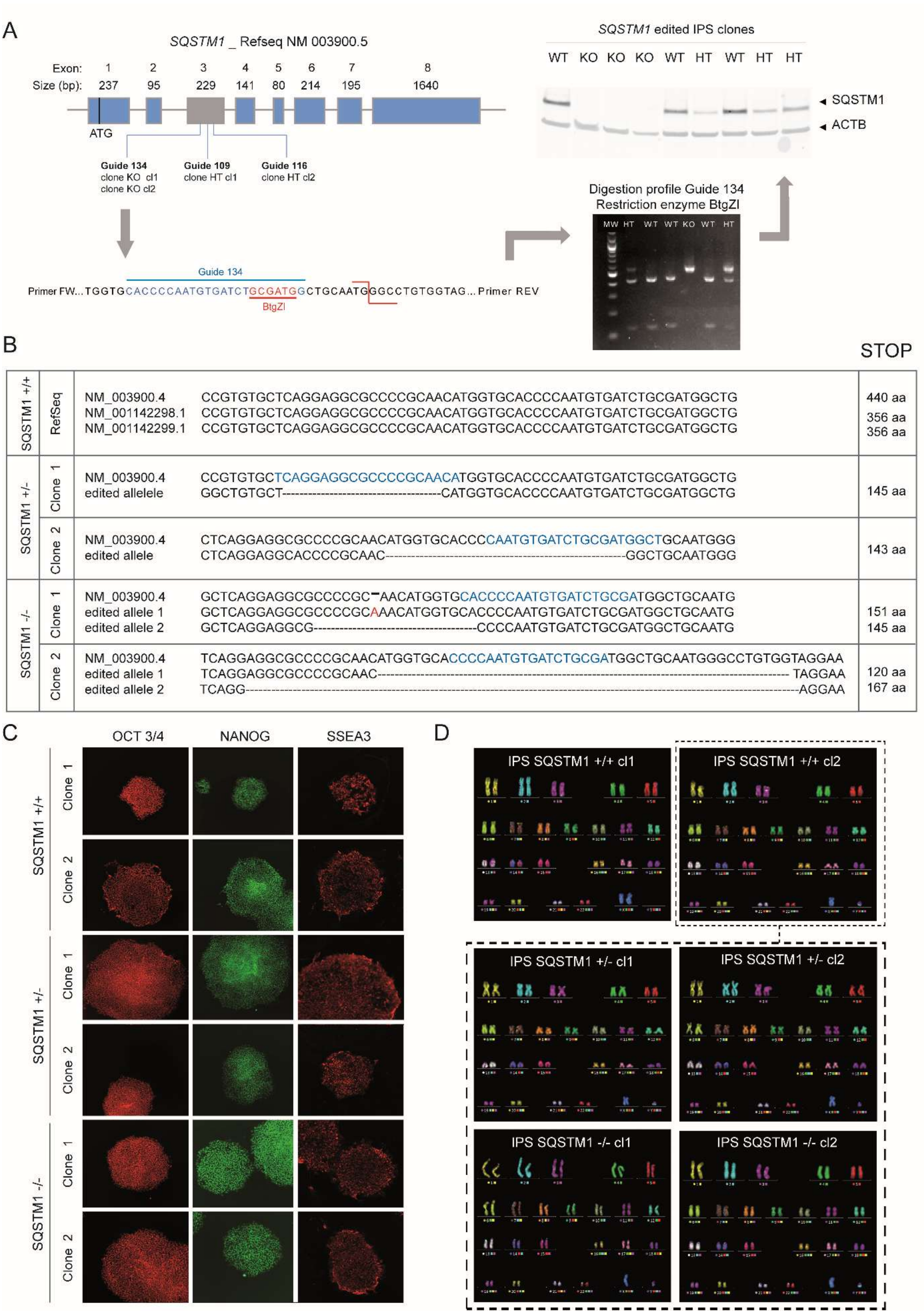
A
SQSTM1 _ Refseq NM 003900.5
Exon: 1 2 3 4 5 6 7 8
Size (bp): 237 95 229 141 80 214 195 1640
ATG
Guide 134
clone KO cl1
clone KO cl2
Guide 109
clone HT cl1
Guide 116
clone HT cl2
Guide 134
Primer FW... TGGTGCACCCCAATGTGATCTGCGATGGCTGCAATGGGCCTGTGGTAG... Primer REV
BtgZI
SQSTM1 edited IPS clones
WT KO KO KO WT HT WT HT HT
SQSTM1
ACTB
Digestion profile Guide 134
Restriction enzyme BtgZI
MW HT WT WT KO WT HT
B
STOP
SQSTM1 +/+
RefSeq
NM_003900.4 CCGTGTGCTCAGGAGGCGCCCCGCAACATGGTGCACCCCAATGTGATCTGCGATGGCTG 440 aa
NM_001142298.1 CCGTGTGCTCAGGAGGCGCCCCGCAACATGGTGCACCCCAATGTGATCTGCGATGGCTG 356 aa
NM_001142299.1 CCGTGTGCTCAGGAGGCGCCCCGCAACATGGTGCACCCCAATGTGATCTGCGATGGCTG 356 aa
SQSTM1 +/-
Clone 1
NM_003900.4 CCGTGTGCTCAGGAGGCGCCCCGCAACATGGTGCACCCCAATGTGATCTGCGATGGCTG
edited allelele GGCTGTGCT-----------------------------------CATGGTGCACCCCAATGTGATCTGCGATGGCTG 145 aa
Clone 2
NM_003900.4 CTCAGGAGGCGCCCCGCAACATGGTGCACCCCAATGTGATCTGCGATGGCTGCAATGGG
edited allele CTCAGGAGGCACCCCGCAAC--GGCTGCAATGGG 143 aa
SQSTM1 -/-
Clone 1
NM_003900.4 GCTCAGGAGGCGCCCCGC-AACATGGTGCACCCCAATGTGATCTGCGATGGCTGCAATG
edited allele 1 GCTCAGGAGGCGCCCCGCAAACATGGTGCACCCCAATGTGATCTGCGATGGCTGCAATG 151 aa
edited allele 2 GCTCAGGAGGCG-------------------------------------CCCCAATGTGATCTGCGATGGCTGCAATG 145 aa
Clone 2
NM_003900.4 TCAGGAGGCGCCCCGCAACATGGTGCACCCCAATGTGATCTGCGATGGCTGCAATGGGCCTGTGGTAGGAA
edited allele 1 TCAGGAGGCGCCCCGCAAC-- TAGGAA 120 aa
edited allele 2 TCAGG---AGGAA 167 aa
C
OCT 3/4
NANOG
SSEA3
SQSTM1 +/+
Clone 1
Clone 2
SQSTM1 +/-
Clone 1
Clone 2
SQSTM1 -/-
Clone 1
Clone 2
D
IPS SQSTM1 +/+ cl1
IPS SQSTM1 +/+ cl2
IPS SQSTM1 +/- cl1
IPS SQSTM1 +/- cl2
IPS SQSTM1 -/- cl1
IPS SQSTM1 -/- cl2

Figure S4. Generation of *SQSTM1* +/- and *SQSTM1* -/- hiPSC clones by the use of CRISPR/Cas9 technology, related to Figure 5.
(A) Schematic representation of the editing strategy targeting Exon 2 of the *SQSTM1* gene. Guide 134 was used to generate two different *SQSTM1* -/- clones from 14c5 hiPSCs, while guides 109 and 116 were employed to obtain *SQSTM1* +/- hiPSC clones 1 and 2, respectively. Edited clones were selected based on their restriction profiles, confirmed by SQSTM1 Western blot analysis.
(B) Sequences of *SQSTM1*-edited hiPSC clones show deletions or insertions on one or both alleles near the guide sequences (highlighted in blue). The position of the predicted premature STOP codon is indicated.
(C) Representative immunocytochemistry images showing pluripotency markers OCT3/4 (red), NANOG (green), and SSEA3 (red) staining in control and edited 1869 hiPSC colonies.
(D) Karyotype analysis of non-edited and edited *SQSTM1* hiPSC clones using mFish. *SQSTM1* +/- and *SQSTM1* -/- clones are derived from the hiPSC control clone 2 (cl2).

	Off-targets SQSTM1 -/- clones 1&2 sgRNA 134	CFD Score
1	intron_B4GALT7_chr5_177603242	0,45
2	intron_FAM184A_chr6_119129291	0,32
3	intergenic_LINC00459|LINC00448_chr13_62397889	0,31
4	intergenic_RP11-420N3.3/RP11-420N3.2|RP11-420N3.2/RP11-420N3.3_chr16_5491023	0,29
5	intron_TESC_chr12_117069990	0,24
6	intergenic_LINC00379|MIR17HG_chr13_91300197	0,24
7	intergenic_NPPA|NPPB_chr1_11854812	0,24
8	intergenic_RP11-403A3.3|LINC00396_chr13_110042906	0,23
9	intergenic_RP11-520A21.1|RP11-372H2.1_chr3_41033547	0,23
10	intron_DIAPH2_chrX_97220513	0,22

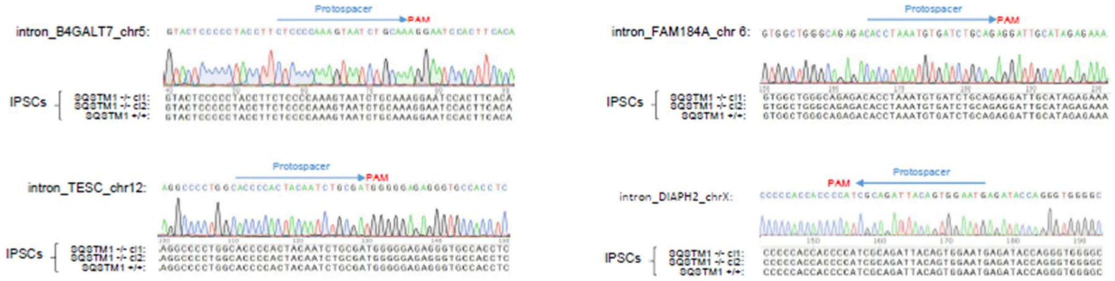

	Off-targets SQSTM1 +/- clone 1 sgRNA 109	CFD Score
1	intergenic_PRICKLE2|RP11-14D22.2/PRICKLE2_chr3_64350556	0.58
2	intron_DNAH17_chr17_78426708	0.25
3	intergenic_LINC00320|NCAM2_chr21_20905590	0.24
4	intergenic_LL09NC01-139C3.1|RP11-145E17.2_chr9_134240780	0.24
5	intergenic_LINC00961|RP11-327L3.3_chr9_35914472	0.20
6	intergenic_CCL1|AC011193.1_chr17_34424483	0.19
7	intergenic_RNA5SP167|RNU6-1230P_chr4_149916252	0.19
8	intergenic_C17orf74|TMEM102_chr17_7432704	0.17
9	intron_RP11-218F4.1_chr17_27963596	0.16
10	intergenic_CTD-2316B1.2|TMEM161B_chr5_88053056	0.13

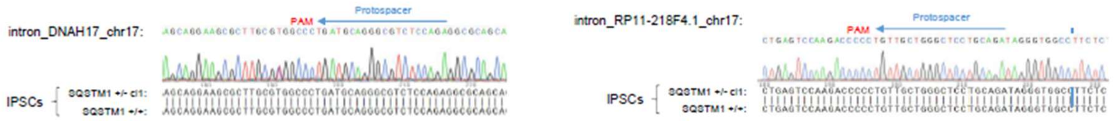

	Off-targets SQSTM1 +/- clone 2 sgRNA 116	CFD Score
1	intergenic_RP11-428L9.2|LINC00709_chr10_9174267	0.69
2	intergenic_GSK3B|AC092910.1_chr3_120041470	0.65
3	intron_CD109_chr6_73750302	0.43
4	intron_MYT1_chr20_64172097	0.42
5	intron_FAM114A1_chr4_38925695	0.38
6	intron_NABP1_chr2_191685192	0.35
7	intergenic_RP11-384F7.1|IGSF11_chr3_118619727	0.31
8	intergenic_RP11-100M12.2|AC104002.1_chr15_27429789	0.26
9	intergenic_RP11-420N3.3/RP11-420N3.2|RP11-420N3.2/RP11-420N3.3_chr16_5491025	0.22
10	intergenic_AL356154.1|NRG3_chr10_81149806	0.21

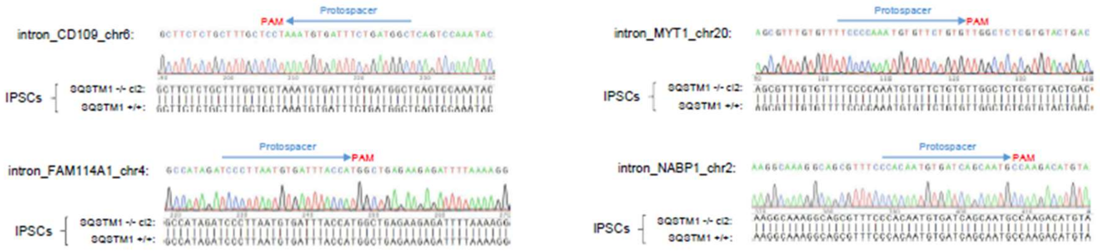

Figure S5. Off target analysis in edited *SQSTM1* +/- and *SQSTM1* -/- hiPSC lines, related to Figure 5. Potential off target edition sites in genomic DNA were identified with the CRISPOR (http://crispor.tefor.net/) software for the three guides used in the study. Region of interest located within genes were amplified by PCR and analyzed by Sanger sequencing. CFD means Cutting frequency determination indicated by the CRISPOR software.

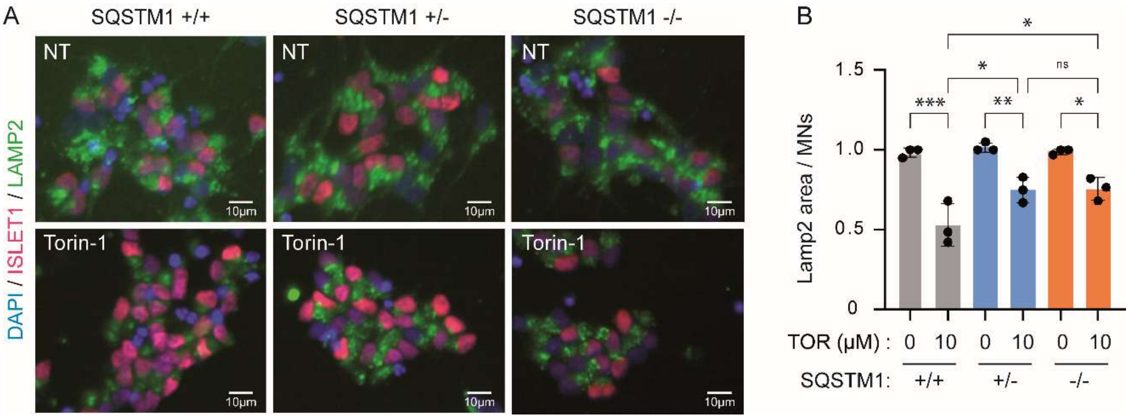

Figure S6. Impact of SQSTM1 loss of expression on LAMP2⁺ lysosomes after Torin-1 treatment in hiPSCs derived MNs, related to Figure 5.

(A and B) Representative immunocytochemistry images (A) and quantification (B) of DAPI (blue), ISLET1 (red), and LAMP2 (green) staining in hiPSC-derived MNs with *SQSTM1*+/+, *SQSTM1*+/-, and *SQSTM1*-/- genotypes treated with 10 µM Torin-1 for 24 hours. Scale bar: 10µm. Plots display the LAMP2 staining area normalized to ISLET1+ MNs, presented as mean ± SD from three independent differentiations from MNs progenitors. Statistical significance was determined using two-way ANOVA with Šídák's post hoc multiple comparisons test.

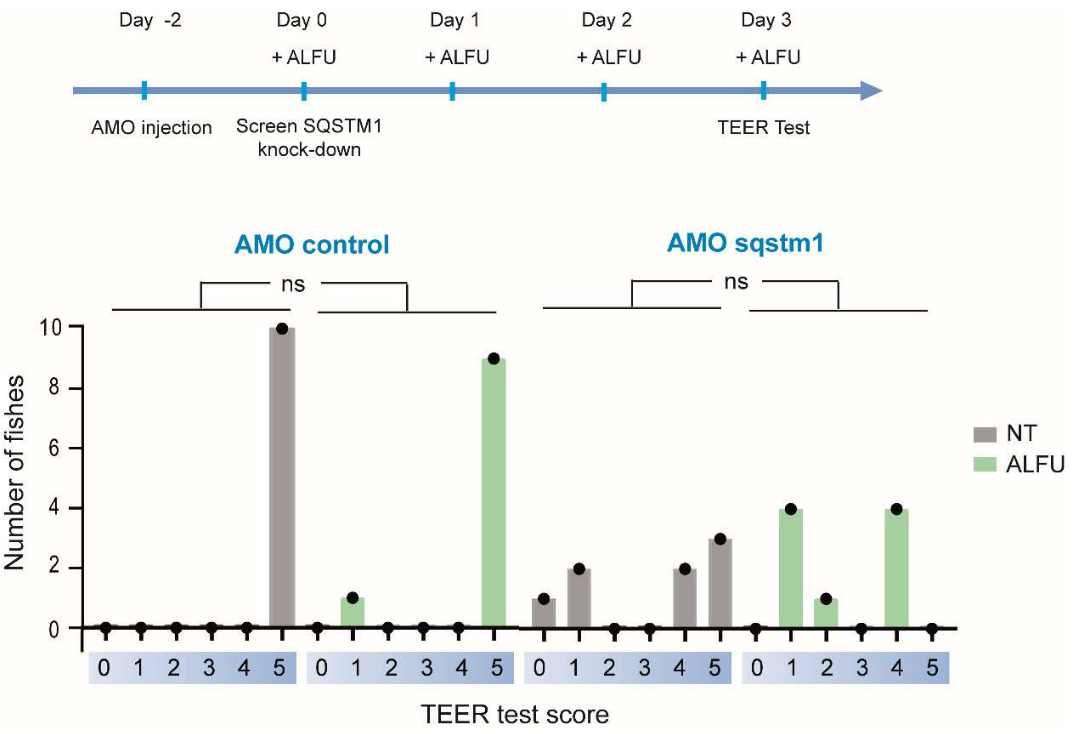

Figure S7. Analysis of alfuzosin treatment on zebrafish with sqstm1 knock-down, related to Figure 7. Quantification of swimming parameters in embryos injected with control or sqstm1-specific AMO, either untreated or treated with alfuzosin for 3 days, using a ViewPoint system. The qualitative scores ranged from 0-5. Data are presented from one experiment on 37 zebrafishes. Statistical significance was determined using a Chi-square test for contingency data. ns: not significative.

Table S1. List of the Drugs tested for their repositioning potential, related to Figure 1.

Drug	Synonyms	Activity	Family/Therapeutic target	Pubchem CID	FDA or EMA-approved	French AMM
Cyproheptadine	Periactine	Anti-Allergic	Serotonin receptor antagonist	2913	+	
Molsidomine	Corvasal	Anti-Anginal	Relaxation in the coronary blood vessels	5353788	-	+
Nicorandil	Ikorel	Anti-Anginal	Arterial and a venous dilator	47528	-	+
Indacaterol	Onbreezhaler	Anti-Asthmatic	Bronchodilator	6918554	+	
Salbutamol	Albuterol	Anti-Asthmatic	Bronchodilator	2083	+	
Levofloxacine	Tavanique	Anti-Bacterial	Fluoroquinolone antibiotic	149096	+	
Manumycin	Manumycin A	Anti-Bacterial	Fatty amide	6438330	-	-
Roxithromycin	Rulid	Anti-Bacterial	Macrolide antibiotic	6915744	-	+
Sulbactam	Unasyn	Anti-Bacterial	Beta-lactamase inhibitor	130313	+	
Tetracycline	Pylera	Anti-Bacterial	polyketide antibiotic	54675776	+	
Tobramycin	Tobradex	Anti-Bacterial	Aminoglycoside antibiotic	36294	+	
Argatroban	Arganova	Anti-Coagulant	Platelet Aggregation Inhibitors	92722	+	
Rivaroxaban	Xarelto	Anti-Coagulant	Inhibitor of the coagulation factor Xa	9875401	+	
Piracetam	Nootropyl	Anti-Convulsant	Neurotransmission promoter	4843	-	+
Amoxapine	Defanyl	Anti-Depressant	Norepinephrine and serotonin re-uptake inhibitor	2170	+	
Doxepin	Quitaxon	Anti-Depressant	Norepinephrine and serotonin-reuptake inhibitor	3158	+	
Fluoxetine	Prozac	Anti-Depressant	Selective serotonin-reuptake inhibitor	3386	+	
Gliclazide	Diamicron	Anti-Diabetic	Insulin secretagogue	3475	-	+
Glyburide	Daonil	Anti-Diabetic	Insulin secretagogue	3488	+	
Pioglitazone	Actos	Anti-Diabetic	Inducer of cellular responsiveness to insulin	4829	+	
Divalproate sodium	Depakote	Anti-Epileptic	Gamma-aminobutyric acid (GABA) transaminase inhibitor	18330669	+	
Fluconazole	Diflucan	Anti-Fungal	Triazole	3365	+	
Ketoconazole	Ketoderm	Anti-Fungal	Phenylpiperazine	456201	+	
Pentamidine	Pentacarinat	Anti-Fungal	Interfering with DNA replication	4735	+	
Terbinafine	Lamisil	Anti-Fungal	Equalene epoxidase inhibitor	1549008	+	
Furosemide	Lasilix	Anti-Hypertensive	Potent loop diuretic	3440	+	
Lisinopril	Zestril	Anti-Hypertensive	Angiotensin-converting enzyme (ACE) inhibitor	5362119	+	
Manidipine	Iperten	Anti-Hypertensive	calcium channel blocker	4008	-	+
Prazosin	Minipress	Anti-Hypertensive	Alpha-1 adrenergic receptor inhibitor	4893	+	
Propranolol hydrochloride	Avlocardyl	Anti-Hypertensive	Beta-adrenergic receptor blocker	62882	+	
Quinapril HCl	Accupril	Anti-Hypertensive	Angiotensin converting enzyme (ACE) inhibitor	54891	+	
Spironolactone	Aldactone	Anti-Hypertensive	Potassium-sparing diuretic	5833	+	
Tadalafil	Cialis	Anti-Hypertensive	Cyclic guanosine monophosphate (cGMP)-specific type 5 phosphodiesterase inhibitor	110635	+	
Urapidil	Eupressyl	Anti-Hypertensive	Adrenergic alpha-1 receptors inhibitor	5639	-	+
Risedronic sodium	Actonel	Anti-Hypocalcemic	Bisphosphonate	5245	+	
Fludrocortisone acetate	Flucortac	Anti-inflammatory	Glucocorticoid-receptor agonist	225609	+	
Indoprofen	Flosint	Anti-inflammatory	Prostaglandin-endoperoxide synthase inhibitor	3718	-	-
Leflunomide	Arava	Anti-inflammatory	Antirhumatismal	3899	+	
Nepafenac	Nevanac	Anti-inflammatory	Cyclooxygenase 1 and 2 inhibitor	151075	+	
Fenofibrate	Lipanthyl	Anti-Lipidemic	Peroxisome proliferator activated receptor alpha (PPARalpha) activator	3339	+	
Rosuvastatin	Crestor	Anti-Lipidemic	Hydroxymethyl-glutaryl coenzyme A (HMG-CoA) reductase inhibitor	446157	+	
Chloroquine	Plaquenil	Anti-Malarial	Inhibit the parasitic enzyme heme polymerase	2719	+	
Zolmitriptan	Zomig	Anti-Migraine	serotonin (5-HT) 1B receptors activator	60857	+	
Esomeprazole	Innexium	Anti-Peptic	Gastric proton pump inhibitor	9568614	+	
Ranitidine	Azantac	Anti-Peptic	Histamine H2-receptor antagonists	3001055	+	
Trospium chloride	Ceris	Anti-Spasmotic	Muscarinic receptors blockade	5284632	+	
Ganciclovir	Virgan	Anti-Viral	Analogue nucleoside	1,35E+08	+	
Ribavirin	Rebetol	Anti-Viral	Viral RNA synthesis inhibitor	37542	+	
Trifluridine	Viroptic	Anti-Viral	thymidylate synthase inhibitor	6256	+	
Dexmedetomidine	Dexdor	Sedative	Alpha-adrenergic agonist	5311068	+	

Table S2. Differentially expressed genes and splicing after drug treatment in MPCs, related to Figure 1.

Drug	DESeq log2FC ≥ 1,45			DESeq log2FC ≥ 0,4			Farline
	DESeq up	DESeq down	Total	DESeq up	DESeq down	Total	
**DMSO**	x	x	x	x	x	x	x
Amoxapine	19	0	19	279	144	423	54
Argatroban	0	0	0	0	1	1	44
Chloroquine	31	1	32	758	383	1141	17
Cyproheptadine	11	0	11	312	89	401	54
Dexmedetomidine	0	0	0	0	0	0	27
Divalproate sodium	0	0	0	0	7	7	73
Doxepin	4	0	4	60	1	61	0
Esomeprazole	0	0	0	0	0	0	30
Fenofibrate	0	0	0	0	0	0	34
Fluconazole	0	0	0	0	0	0	31
Fludrocortisone acetate	3	0	3	108	162	270	31
Fluoxetine	17	0	17	239	93	332	32
Furosemide	0	0	0	0	0	0	47
Ganciclovir	0	0	0	0	0	0	15
Gliclazide	0	0	0	0	0	0	46
Glyburide	0	0	0	0	9	9	53
Indacaterol	24	0	24	385	132	517	47
Indoprofen	0	0	0	0	7	7	29
Ketoconazole	11	0	11	143	21	164	27
Leflunomide	0	0	0	25	38	63	21
Levofloxacine	0	0	0	0	0	0	23
Lisinopril	0	0	0	0	0	0	12
Manidipine	2	0	2	145	68	213	45
Manumycin	236	217	453	1508	1517	3025	125
Molsidomine	0	0	0	0	0	0	31
Nepafenac	0	0	0	0	3	3	36
Nicorandil	0	0	0	0	0	0	18
Pentlseth	124	128	252	1459	1446	2905	206
Pioglitazone	0	0	0	0	0	0	21
Piracetam	0	0	0	0	0	0	22
Prazosin	138	15	153	1418	1209	2627	70
Propranolol hydrochloride	0	0	0	0	0	0	27
Quinapril HCl	0	0	0	0	0	0	20
Ranitidine	0	0	0	0	0	0	10
Ribavirin	0	0	0	24	38	62	75
Risedronic acid	0	0	0	0	0	0	25
Rivaroxaban	0	0	0	7	11	18	48
Rosuvastatin	182	306	488	1724	1947	3671	140
Roxithromycin	0	0	0	17	3	20	17
Salbutamol	0	0	0	6	1	7	27
Spironolactone	0	0	0	46	41	87	51
Sulbactam	0	0	0	0	0	0	0
Tadalafil	0	0	0	1	0	1	44
Terbinafine	0	0	0	0	0	0	16
Tetracycline	0	0	0	0	0	0	47
Tobramycin	0	0	0	0	0	0	21
Trifluridine	3	0	3	348	59	407	57
Trospium chloride	0	0	0	0	0	0	34
Urapidil	0	0	0	0	0	0	49
Zolmitriptan	0	0	0	0	8	8	42

Table S3. List of the primers and guides used in the study, related to Figure 1, Figure 2, Supplemental Figure 2, Figure 3, Figure 5 and Figure 7.

Primers for Gene expression analysis		
Gene	Primer Forward	Primer Reverse
PMP22	GCTCCTCCTGTTGCTGAGTA	ATCGACAGGATCATGGTGGC
EGR2	ACCGCCTCCTCCTCCTTATT	GGGTAGGCCAGAGAGGAAGA
DES	ATTGGAGGACCGATTTGCC	TCACCGTCTTCTTGGTATGGA
LMNB1	AGCTGCAAATCTGATGGCCT	GAGGAACCCCTTCCGAACAG
SOD1	TTGGGCAAAGGTGGAAATGAA	CACCACAAGCCAAACGACTT
GDAP1	GACTCCATGATCCCGGCTTA	ACCTGATCCAAGACTTTCTCCA
SQSTM1	AGGACAAATTGCGCCCATTT	TCTCTTTCAGGGACAGGCTG
PLP1	TGAAGCCCTAACTCAGCCAA	AGGGCCATCTCAGGTTACAC
18S	GAGGATGAGGTGGAACGTGT	TCTTCAGTCGCTCCAGGTCT
HSPB8	AAATGTTAGAGGGTGCGGG	TGGCCTAACACAACCCAAGCA
HSPA1A	AGCTGGAGCAGGTGTGTAAC	CAGCAATCTTGGAAAGGCCC
HSPA1B	AGC TGG AGC AGG TGT GTA AC	GAG TCC CAA CAG TCC ACC TC
HSPA5	GAACGTCTGATTGGCGATGC	ACCACCTTGAACGGCAAGAA
HSPH1	AGCAGAAATGAAGTTAGTGTCCA	TGTTTTCTTCTGGGTGGCAAG
OPTN	GACCAGCAGGCTTACCTTGT	GGGGCAGGAATGAATCGGAA
UBC	AACAACAACTGCGACACCCAAA	GGAACAGGCGAGGAAAAGTA
MAP1LC3B	CCGCACCTTCGAACAAAGAG	TTGAGCTGTAAGCGCCTTCT
ITGB4	AATGCAGCCGGTCTGACTC	GTCCTGGACCTACCTCGGG
MMP1	CTGCTGGCAAAACATCCCTT	CGCTTTGATGGCATTGAGGA

Primers for Offtarget analysis in SQSTM1 edited iPSC clones				
SQSTM1 edited clone	Guide Sequences	off target	Forward Primer	Reverse Primer
SQSTM1 -/- clones 1&2 sgRNA 134	TCAGGAGGCGCCCCGCAACA TGG	intron_B4GALT7_chr5_177603242	TCGCTGACTCTTAAGATTGTGA	CGCTCCTCTGGAAGTTCCTG
		intron_FAM184A_chr6_119129291	ACCTATAACCTGGAAGCCCC	CCCACCACATCTGCTTCATG
		intron_TESC_chr12_117069990	TGGCACATCTTGGAAGCTG	AAGCCTGTGGTTCTGAATGA
		intron_DIAPH2_chrX_97220513	CTGTTCCACCATCTGCTCCC	ATTTTACCACGGCCCCTCTG
SQSTM1 +/- clones 1 sgRNA 109	AGCCATCGCAGATCACATTG GGG	intron_DNAH17_chr17_78426708	AGAGAGCCGTGGACAGATCT	CTCAAGGAGCTGAACCTGGG
		intron_RP11-218F4.1_chr17_279635	AAGTGGGGCCTGCATAAGTC	TGCAGGAAAGATGGGCTGAG
SQSTM1 +/- clones 2 sgRNA 116	CACCCCAATGTGATCTGCGA TGG	intron_CD109_chr6_73750302	AGCTCAGTTTGGGTGAGTGG	ATATTGCTGCTGCCTCCACC
		intron_MYT1_chr20_64172097	CTCTTCCTGGAGAGCCCTCT	AGAAGGGAGAGAGCTCGAGG
		intron_FAM114A1_chr4_38925695	AAATCCAGGAGCAATCCAGC	CTGCAACAAACGTGAAAGTGT
		intron_NABP1_chr2_191685192	TGACTCGAAGGCTACATGTCT	CCTTGATTCAGGGCCAGTGT
